# Lingual Taste Nerve Transection Alters Food Selection, Relative Macronutrient Intake, and Meal Patterns in Rats Consuming a Cafeteria Diet without Changing Total Energy Intake

**DOI:** 10.1523/ENEURO.0393-23.2024

**Published:** 2024-03-01

**Authors:** Carolina R. Cawthon, Ginger D. Blonde, Alan C. Spector

**Affiliations:** Department of Psychology, Program in Neuroscience, Florida State University, Tallahassee, Florida 32306

**Keywords:** cafeteria diet, chorda tympani nerve, food choice, glossopharyngeal nerve, meal patterns, taste

## Abstract

The control of ingestive behavior is complex and involves input from many different sources, including the gustatory system. Signals transmitted via the taste nerves trigger responses that promote or discourage ingestion. The lingual taste nerves innervate 70% of taste buds, yet their role in the control of food selection and intake remarkably remains relatively underinvestigated. Here we used our custom five-item Food Choice Monitor to compare postsurgical behavioral responses to chow and a five-choice cafeteria diet (CAF) between male rats that had sham surgery (SHAM) or histologically verified transection of the chorda tympani and glossopharyngeal nerves (2NX). Compared with SHAM rats, 2NX rats ate significantly more of the high-fat CAF foods. The altered food choices led to dramatically increased fat intake and substantially reduced carbohydrate intake by 2NX vs SHAM rats. Furthermore, whether offered chow or CAF, 2NX rats ate fewer, larger meals each day. Eating rates implied that, compared with SHAM, 2NX rats were equally motivated to consume CAF but less motivated to eat chow. Even with these differences, energy intake and weight gain trajectories remained similar between SHAM and 2NX rats. Although some rats experienced CAF before surgery, contrary to our expectations, the effects of prior CAF experience on postsurgical eating were minimal. In conclusion, although total energy intake was unaffected, our results clearly indicate that information from one or both lingual taste nerves has a critical role in food selection, regulation of macronutrient intake, and meal termination but not long-term energy balance.

## Significance Statement

Although it is uncontested that taste guides ingestive behavior, its role in food selection and daily macronutrient consumption remains understudied. Here we transected the gustatory nerves innervating the tongue in rats that had access to a choice of five foods that varied in fat and sugar content. The neurotomy altered food choices, substantially shifting the macronutrient profile of daily intake favoring fat over carbohydrate despite having no effect on overall energy consumption. The nerve transection led to animals eating fewer and larger meals each day, apparently blunting meal termination processes. Thus, the signals in the lingual gustatory nerves significantly contribute to which foods are consumed and how they are eaten.

## Introduction

The control of ingestive behavior is complex and involves signals from many different sources. Immediately before food is swallowed, it contacts oronasal chemoreceptors which send a chemical analysis to the brain, triggering responses that promote or discourage ingestion. In this sense, the gustatory system is key, but there is surprisingly little research investigating how the information arising from taste receptors affects eating, drinking, food selection, and ultimately nutrition.

Cranial nerves (CN) VII, IX, and X transmit taste information to the brain. The greater superficial petrosal (GSP) branch of CN VII innervates taste buds found on the palate. Taste buds of the anterior and posterior tongue are innervated by the chorda tympani (CT) branch of CN VII and the glossopharyngeal nerve (GL), respectively. The remaining taste buds reside mostly in the laryngeal epithelium, are innervated by the superior laryngeal branch of the vagus, and thought to be primarily involved in protection of the airways (Spector, 2003). Notably, over 70% of taste buds are found on the tongue and innervated by either the CT or GL. Decades of research suggest that the different gustatory nerves do not contribute equally to all taste functions including detection and discrimination, oromotor and somatic reflexes (called *taste reactivity*), and approach and avoidance (Spector, 2003; Spector and Glendinning, 2009). Indeed, the effects of single or combined gustatory nerve transection on taste-related behavior vary and depend on the taste stimulus, task, and nerves cut (Spector, 2003).

While the literature is replete regarding the effects of gustatory neurotomy on basic taste functions, the role of these taste nerves on *ad libitum* food choices and intake patterns remains relatively understudied despite their functional significance. Smith et al. (1988) reported that within-meal eating rate was lowered by transection of the CT (CTX) but total powdered chow (PC) intake was unchanged. Even denervating all taste buds of the tongue by transecting the CT and GL together (2NX) does not appear to affect total intake because, at least in our hands, rats quickly recover their presurgical body weights (St. John and Spector, 1998; Blonde et al., 2006). More surprisingly, 2NX has relatively minor effects on preference/aversion functions measured in long-term two-bottle intake tests across a range of concentrations of some prototypical tastants delivered in solution (Grill and Schwartz, 1992). The one exception to this was a study by Treesukosol et al. (2010) who found that, while transection of the GL (GLX) alone modestly affected glucose intake, it strikingly decreased corn oil preference (vs water) and intake via reductions in drinking bout size not number.

Not surprisingly, massive transection of the CT, GL, and GSP caused severe decreases in oil-chow mash and sweetened-milk diet intake and body weight that partially recovered over time (Dotson et al., 2012). The decreased energy intake was driven by meal number, not meal size. Jacquin (1983) reported that 2NX combined with transection of the pharyngeal branch of the vagus caused a prolonged period of decreased intake and body weight that varied as a function of diet characteristics. However, the pharyngeal branch of the vagus is a nongustatory motor nerve involved in swallowing, making it difficult to entirely dissociate sensory from motor effects. Another caveat is that the completeness of the nerve transections and lack of regeneration was not histologically verified.

What is lacking in the gustatory neurotomy studies heretofore conducted in preclinical models is how the histologically confirmed removal of selective gustatory nerves affects eating and selection of a nutritionally optimal diet in the context of multiple food choices. Arguably, the gustatory system evolved, in part, to perform such a function given the complex and diverse nature of macro- and micronutrient sources in the environment. Accordingly, here, rats that had 2NX or sham surgery were given a cafeteria diet of five solid foods with varying sugar and fat content. We used specialized cages to monitor and quantify eating behavior and food selection over 8 d. We show that transection of the lingual gustatory nerves has striking effects on relative macronutrient ingestion and meal patterns despite having no effect on total energy intake.

## Materials and Methods

### Subjects

Subjects for this experiment were 32 male Sprague Dawley rats, ∼7 weeks old on arrival at our facility. Rats were allowed ≥7 d to acclimate to handling and their surroundings before any experimental procedures began. Rats were housed in regular or modified standard polycarbonate tub cages with wood chip bedding in a vivarium with automatic temperature, humidity, and light control that maintained a 12 h light/dark cycle. Unless noted otherwise, rats had *ad libitum* access to chow (either pelleted, powdered, or made into mash by the addition of deionized water) and deionized water throughout the experiment and enrichment was provided by a Rattle-A-Round (Otto Environmental), a custom stainless-steel nest, and/or multiple food choices. All animal procedures were approved by the Florida State University Animal Care and Use Committee.

### Surgery and postoperative care

We used aseptic methods to prepare instruments and perform bilateral nerve transection surgeries, which have been previously described (Spector et al., 1990, 1993, 1996; Spector and Grill, 1992; Breslin et al., 1993, 1995; St. John et al., 1994, 1997a,b, 2003; St. John and Spector, 1996; Markison et al., 1999; Eylam et al., 2000; King et al., 2000; Kopka and Spector, 2001; Geran et al., 2002; Blonde et al., 2006, 2010; Treesukosol et al., 2010; Dotson et al., 2012). Briefly, for the CTX + GLX (2NX), rats were anesthetized with isoflurane (≤5% in 1 L O_2_/min) and positioned in an earbar-less head holder. A midline incision was made to the ventral neck, and the sublingual and submaxillary salivary glands were carefully retracted. The sternohyoid, omohyoid, and posterior belly of the digastric muscle were then retracted and connective tissue dissected to expose the GL, which was cut with microscissors to remove as much as possible, typically ∼10 mm; this has been shown to discourage regeneration (King et al., 2000). The incision was closed with nonabsorbable sutures. Then, the rat was rotated to one side and the external ear canal was retracted to expose the tympanic membrane, which was removed to allow transection of the CT followed by removal of the ossicles. Any remaining tympanic membrane and a portion of the adjacent ear canal were cauterized to promote cerumen production, reducing the chances of CT regeneration. For the SHAM surgery, the GL was visualized but left undisturbed and the external ear canal was carefully retracted to fully expose the tympanic membrane but the membrane was not disturbed to avoid possible damage to the CT. Perioperatively and for 3 d after surgery, rats received carprofen (5 mg/kg body weight, s.c.) for control of postsurgical pain and gentamicin (8 mg/kg body weight, s.c.) to prevent infection. In addition to *ad libitum* standard chow, all rats received a wet mash made from powdered rodent chow and deionized water for at least 3 d after surgery, or until body weight returned to ≥90% presurgical weight. Sutures were removed 7–10 d after surgery. One rat failed to regain weight after surgery and was euthanized.

### Apparatus

**Figure 1.**
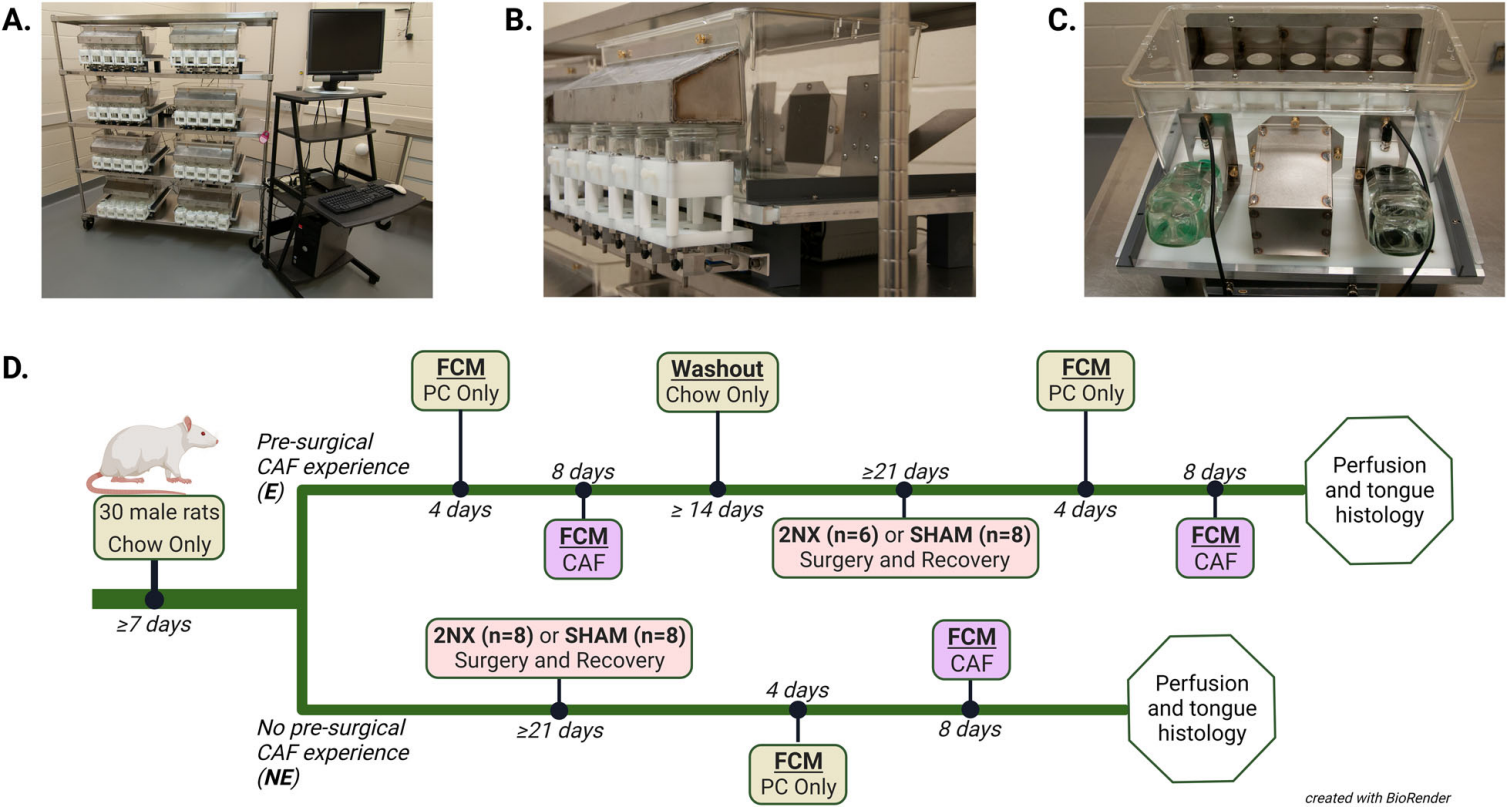
FCM and experimental timeline. ***A***, Eight FCM cages with PC controller. ***B***, Five food jars with FCM cage. ***C***, View of food access hood, water bottles, and nest on FCM cage. ***D***, Experimental timeline. PC, powdered chow; CAF, five-choice custom rodent diet; 2NX, combined CT + GL transection.

At designated times during this study, the rats were placed into our custom five-item Food Choice Monitor (FCM, Fig. 1*A–C*), thoroughly described by Blonde et al. (2022). Briefly, the FCM provides continuous recording of changes in weight of up to five food jars and licks from two fluid bottles allowing a detailed analysis of what, when, and how much rats eat and drink during each recording period, typically 20–22 h daily. The system uses standard polycarbonate rat cages with modifications to allow access to a linear array of five food jars via a stainless-steel hood with five separated compartments that enable rats to eat while preventing mixing of foods. Food jar weight changes are measured 10 times per second and collected in 10 s bins. Opposite the feeding hood are two lick blocks, each of which houses a drinking spout attached to a fluid bottle. An electrical contact circuit continuously reports licking on the drinking spouts which is time-stamped. Between the water bottles is a nest that provides environmental enrichment for the animal. In this experiment, intake measurements occurred for 22 h daily, with the remaining 2 h per day for animal care and FCM maintenance. During all monitoring phases of this study, the 2 h maintenance period occurred from ∼8:30 to 10:30 A.M. each day (beginning 0.5 h into the light period and ending at 2.5 h into the light period). To prevent positional bias, positions of food jars and fluid bottles were rotated daily. In addition to FCM recordings, food jars and water bottles were manually weighed before and after daily replacement to provide external validation of recorded intake measures. For this experiment, minimum meal size was set to 1 kcal and a pause in consumption of ≥900 s (15 min) was used to separate meals (Blonde et al., 2022).

### Diets

The rats were given a cafeteria-style 5-Choice Custom Rodent Diet Array (CAF) during 8 d periods of meal pattern monitoring. The choices consisted of four custom rodent diets (Research Diets) varying in fat and sugar content and powdered standard rodent chow as the fifth choice. Macronutrient profiles and energy densities for each food choice are provided in Table 1. The custom rodent diet choices were identical in the content of vitamins, minerals, protein, and fiber. The total fat in the custom rodent choices consisted of 10% soybean oil and 90% lard. All sugar in the custom rodent diets was sucrose and nonsugar carbohydrate was cornstarch. The diet design aimed to make the diets as similar as possible in all but sugar and fat content. All custom rodent choices were found to be preferred over PC (Cawthon and Spector, 2023).

**Table 1.** Five-choice custom rodent diet array (CAF)

Choices	Research diets #	% CHO	% SUG	% PRO	% FAT	kcal/g
PC	n/a	57.5	9.2	28.9	13.6	3.35
LFLS	D21102802	67.0	4.0	20.0	13.0	3.79
LFHS	D21102803	67.0	66.5	20.0	13.0	3.79
HFLS	D21102804	10.0	4.0	20.0	70.0	5.41
HFHS	D21102801	25.4	24.8	20.0	54.6	4.85

Nutritional characteristics of CAF shown as percentage of kcal. Food choices were powdered chow (PC) or custom rodent diets that were (L) or high (H) in sugar (S) and/or fat (F) and). CHO, carbohydrate; SUG, sugar; PRO, protein.

### Experimental design

The experimental design is depicted in Figure 1*D*. After at least 7 d of acclimation to our facility, the rats were divided into two diet experience groups. One group was designated to have and the other not to have experience with CAF prior to nerve transection surgery. After acclimation, the experienced group (E) was placed into the FCM and received 4 d of only PC followed by 8 d of access to CAF. After this FCM session, experienced rats had a 16 d washout period during which they had only standard chow. Then, experienced rats were evenly divided to have either 2NX or sham (SHAM) surgery, with groups matched as closely as possible based on body weight and intake during the presurgical FCM session. The washout period was included to isolate weight loss resulting from removal of CAF from that resulting from surgery, thus allowing for better postoperative monitoring. After a minimum of 18 d for recovery, the experienced rats were returned to the FCM for a second 12 d monitoring period beginning with 4 d of only PC followed by 8 d with CAF. The rats with no presurgical diet experience (NE) were separated into surgical groups matched as closely as possible based on body weight and received 2NX or SHAM. After at least 15 d for recovery from surgery, the rats were placed into the FCM for 4 d with only PC followed by 8 d with CAF. Rats in all groups were perfused the day after completing the postsurgical CAF exposure, and tongues were collected for histological verification of nerve transections.

### Histology

The day after completing the postsurgical CAF exposure, rats were deeply anesthetized with 0.5 ml pentobarbital sodium (Euthasol, 390 mg/ml pentobarbital sodium, i.p.) and then transcardially perfused with saline followed by 10% buffered formalin. The tongue was removed and stored in 10% formalin until further processing. The anterior tongue was separated from the posterior tongue just anterior to the intermolar eminence. The anterior portion was dipped in 0.5% methylene blue for 30–60 s and then immediately rinsed in deionized water. The tissue was then split medially and muscle removed from each half such that the epithelium could lay flat between two glass slides. Each half was viewed using light microscopy and fungiform papillae with and without intact taste pores counted. The percentage of papillae with intact pores was calculated to assess completeness of CT transections, and rats with <25% of anterior tongue papillae having pores were deemed complete transections. This cutoff is based on functional assessments in rats with regenerated CT (Kopka et al., 2000). The posterior portion of the tongue containing the circumvallate papillae (CV) was embedded in paraffin and sliced on a microtome into 10-µm-thick sections. Hematoxylin and eosin staining was performed so that intact taste buds could be identified and used to assess the completeness of GL transections. The CVs were first qualitatively assessed to identify tissue that had many taste buds, presumably reflecting the absence of transected nerves or substantial regeneration, from tissue that had only a few, presumably reflecting animals with transected nerves. The taste buds with taste pores in the CV from presumably nerve-transected animals were then counted. Rats with fewer than 30 taste buds, a value used previously by our laboratory (Treesukosol et al., 2010), were considered to have successful GLX without significant regeneration. In a couple of cases, missing slices from the CV or problems with the tissue resulted in <100% of a taste bud field being counted. We cannot rule out that intact buds in the missing or damaged sections were overlooked, but the undamaged sections had none. The tissues were coded so that the observer assessing the taste buds was unaware of group assignment.

### Data analysis

All statistical analyses were performed using Systat 13 (Inpixon). Body weight within each experience group was compared using two-way ANOVA (surgery × day) during three periods: the 7 d immediately preceding the surgery week; the 7 d represented by Days 3–9 after completion of surgeries; the 8 d CAF diet period. For intake and meal parameters, the 4 d PC period was considered separately from the 8 d CAF period in three-way ANOVAs (surgery × experience × day). Intake and meal parameters were also compared on the first and last day of the diet phases (i.e., PC Day 1, PC Day 4, CAF Day 1, CAF Day 8) using two-way ANOVA (surgery × experience). Meal energy density was calculated by dividing the average meal kcal by the average meal mass (g). Statistical results were considered to be significant when *p* ≤ 0.05. The final group sizes used in all analyses were as follows: SHAM with CAF experience (SHAM-E), *n* = 8; 2NX with CAF experience (2NX-E), *n* = 6; SHAM without CAF experience (SHAM-NE), *n* = 8; 2NX without CAF experience (2NX-NE), *n* = 8.

## Results

### Nerve transections were histologically verified

**Figure 2.**
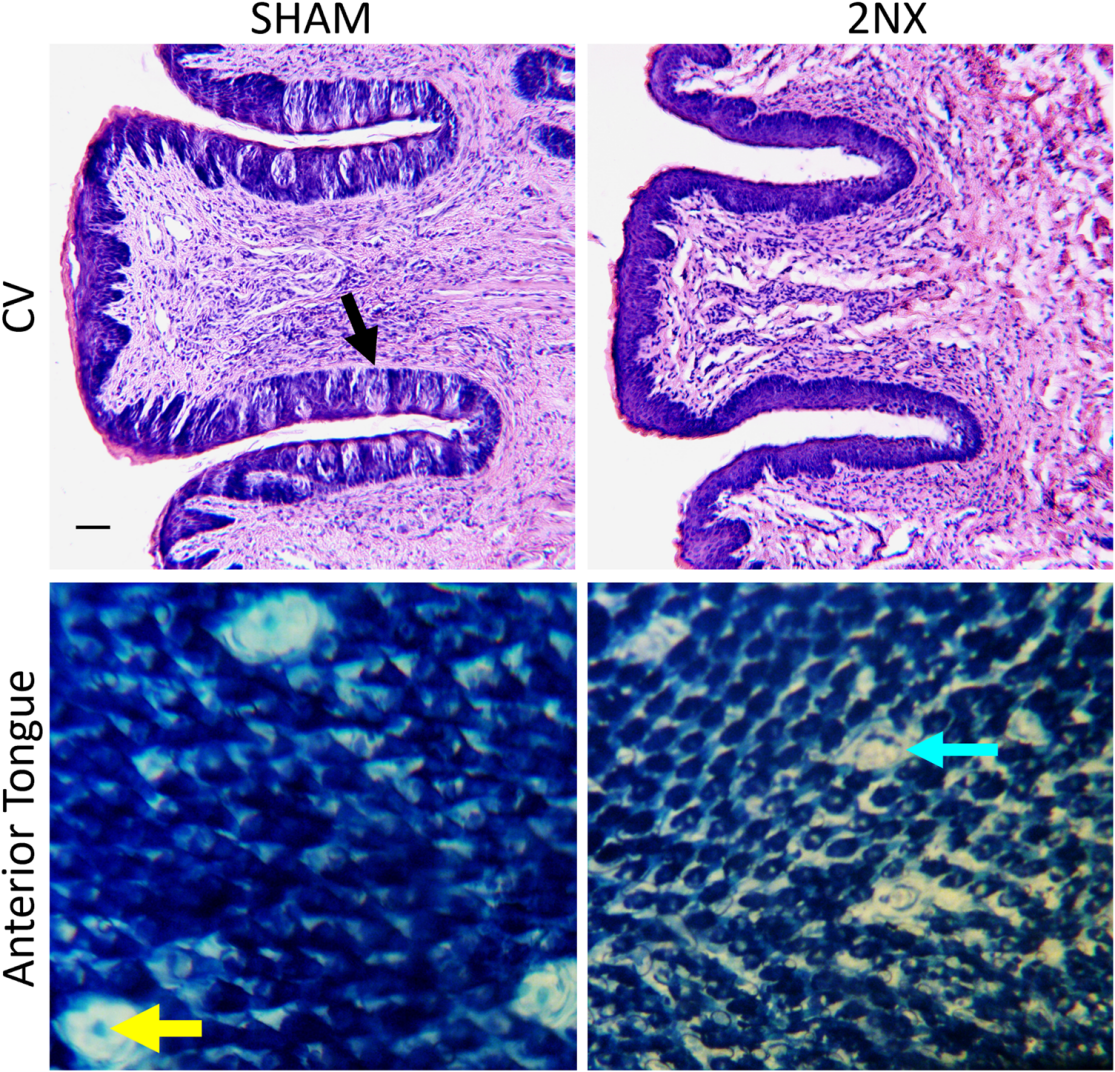
Representative tongue histology reveals differences between SHAM and 2NX rats. Representative images of circumvallate (CV, top) slices and a portion of anterior tongue surface (bottom) from a SHAM (left) and a 2NX (right) rat. The SHAM animal has many taste buds in the CV (top left); a taste bud with a typical taste pore is shown at black arrow. The fungiform papillae of the anterior tongue of the SHAM rat (top right) also have pores, stained as a blue dot in the center of each papilla (yellow arrow). The 2NX CV lacks taste buds (top right) and the fungiform papillae are smaller and lack pores (bottom right, aqua arrow). For the CV, all available tissue was viewed. Taste pores were quantified only in 2NX animals as the difference between surgical groups is readily apparent. Photomicrographs were adjusted for contrast and brightness. For the CV images, scale bar is 50 µm.

All but one rat that had 2NX were verified as meeting our histological criteria for complete nerve transection with absence of significant regeneration. Representative images (Fig. 2) reveal the dramatic differences in tongue histology between SHAM and 2NX rats. Transected rats had 4.35 ± 1.48% (mean ± SE) of anterior tongue papillae with pores compared with SHAM rats which had 95.9 ± 0.6% (mean ± SE). For the posterior tongue, 2NX rats had no more than four intact taste buds while, although only qualitatively evaluated, SHAM rats had *many* more. One rat from the 2NX-E was excluded from all data analyses because it was found to have >25% of papillae with pores on one side of the anterior tongue.

### Lingual gustatory neurotomy did not alter weight gain trajectory or total energy intake

**Figure 3.**
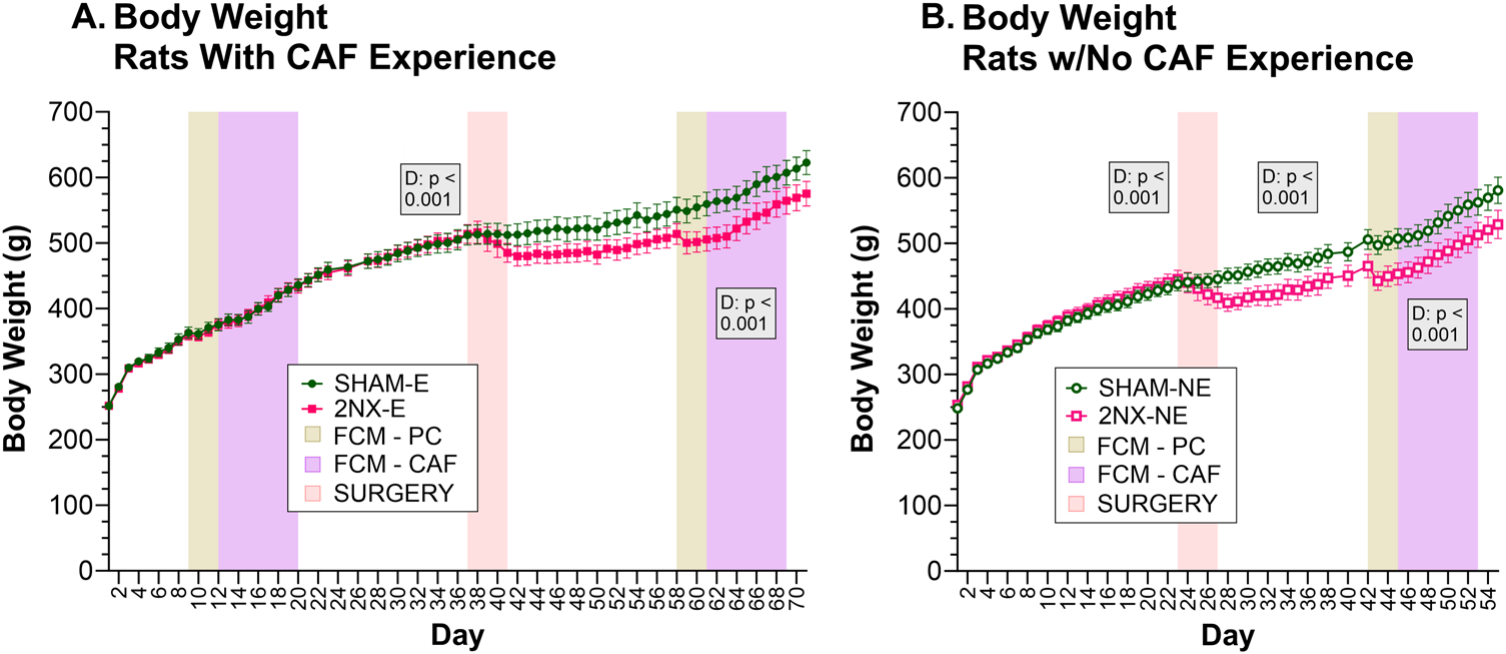
Weight gain trajectory was not altered by 2NX. Graphs depict MEAN ± SE body weight for rats in each surgical group. Due to a slight offset in the experimental timeline of rats with and without presurgical CAF diet experience, the experience groups were separated for two-way repeated-measures ANOVAs for key periods: 7 d immediately before surgeries began, Days 3–9 after completion of surgeries, and the 8 d of CAF meal pattern and intake monitoring in the FCM. Group sizes: SHAM-E, *n* = 8; 2NX-E, *n* = 6; SHAM-NE, *n* = 8; 2NX-NE, *n* = 8. Significant effects of day (D) or surgery (S) or interactions between factors are indicated in the shaded boxes and complete statistical details can be found in Table 2. ***A***, Body weight of rats with CAF diet experience prior to 2NX or SHAM surgery. ***B***, Body weight of rats that did not have presurgical CAF diet experience.

A comparison of body weights within each diet experience condition (E and NE) confirmed that rats were similar prior to surgery (Fig. 3, Table 2). The lack of weight difference is clearly seen by the overlap of mean group weights during the presurgical phases in Figure 3. Regardless of diet experience, rats that had 2NX lost more weight after surgery than SHAM controls and the group mean weight curves of 2NX rats remained visibly offset from that of their SHAM counterparts for the remainder of the experiment. However, comparison of body weights between the respective surgical groups on Days 3 through 9 after completion of surgeries failed to reach statistical significance and the lack of an interaction between surgery and day indicates that the 2NX and SHAM groups were gaining weight along the same trajectory. These results remained true for the CAF phase (Fig. 3, Table 2).

**Table 2.** Body weight statistics

Effects	7 d Pre-SX 2NX-E vs SHAM-E	Days 3–9 after all SX 2NX-E vs SHAM-E	During CAF 2NX-E vs SHAM-E
Surgery	*F*_(1,12)_ = 0.010, *p* = 0.922	*F*_(1,12)_ = 2.543, *p* = 0.137	*F*_(1,11)_ = 3.813, *p* = 0.077
Day	***F*_(6,72)_ = 50.156, *p* < 0.001**	*F*_(6,72)_ = 1.547, *p* = 0.175	***F*_(7,77)_ = 105.031, *p* < 0.0001**
S × D	*F*_(6,72)_ = 0.395, *p* = 0.880	*F*_(6,72)_ = 0.508, *p* = 0.801	*F*_(7,77)_ = 1.863, *p* = 0.087
Effects	7 d Pre-SX 2NX-NE vs SHAM-NE	Days 3–9 after all SX 2NX-NE vs SHAM-NE	During CAF 2NX-NE vs SHAM-NE
Surgery	*F*_(1,14)_ = 0.264, *p* = 0.616	*F*_(1,14)_ = 4.393, *p* = 0.055	*F*_(1,14)_ = 4.223, *p* = 0.059
Day	***F*_(6,84)_ = 205.564, *p* < 0.001**	***F*_(6,84)_ = 25.549, *p* < 0.001**	***F*_(7,98)_ = 177.922, *p* < 0.001**
S × D	*F*_(6,84)_ = 0.623, *p* = 0.712	*F*_(6,84)_ = 0.686, *p* = 0.661	*F*_(7,98)_ = 0.443, *p* = 0.873

Results comparing body weight between surgery groups and within each diet experience group during the 7 d period prior to surgeries beginning, on Days 3–9 after surgeries were completed, and while on CAF (2-way ANOVA). Effects and interactions are defined as follows: S, surgery; D, day. Statistics with *p* value ≤0.05 are bolded.

**Figure 4.**
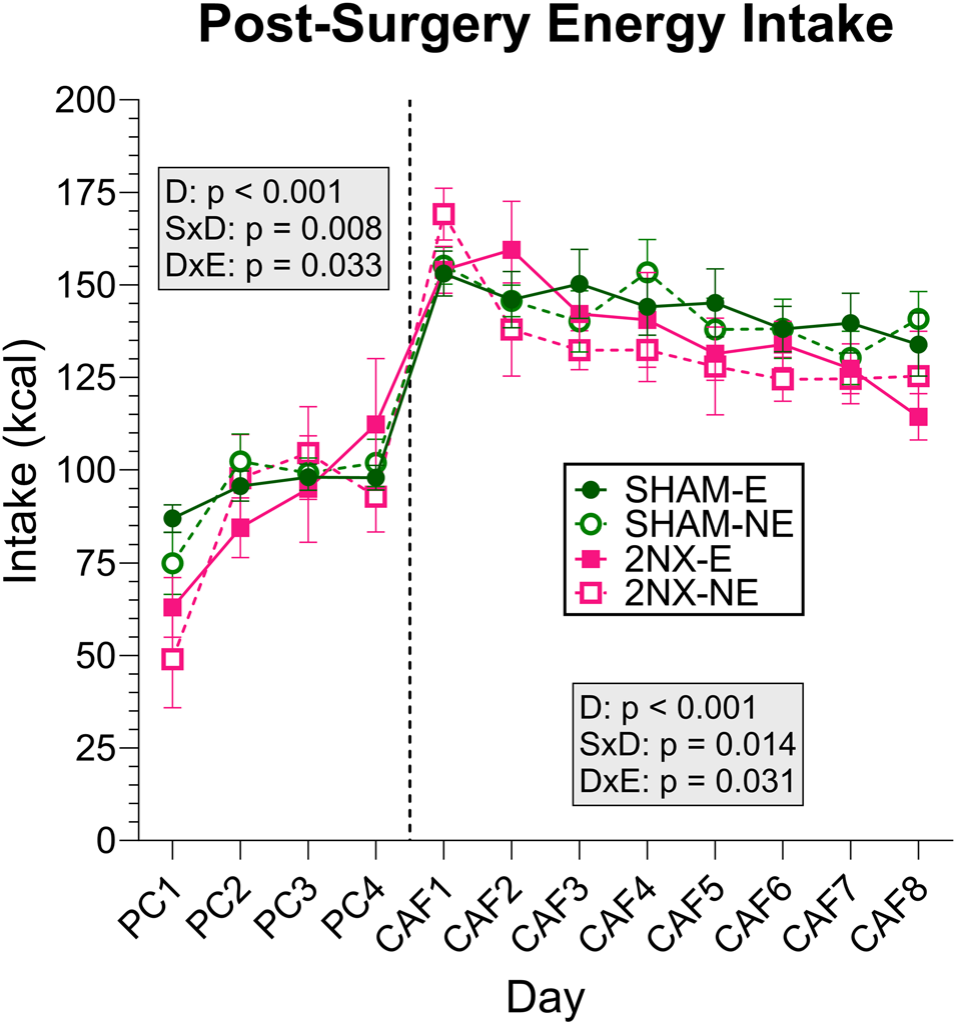
Energy intake is similar between rats that had 2NX and SHAM surgery. Postsurgery energy intake represented as mean ± SE. Rats that had SHAM surgery are shown as circles and 2NX as squares, and rats with presurgical CAF diet experience (E) are shown with filled symbols and without experience (NE) with open symbols. The vertical line separates the four PC (powdered chow) days from the eight CAF days, which were analyzed separately with three-way ANOVA. Significant effects of surgery (S), CAF diet experience (E), and day (D) or interactions between factors are indicated in the shaded boxes for the PC and CAF diet phases. Group sizes: SHAM-E, *n* = 8; 2NX-E, *n* = 6; SHAM-NE, *n* = 8; 2NX-NE, *n* = 8. Statistical details can be found in Tables 3 and 4.

We then compared total intake for each diet phase using three-way ANOVAs and found no main effects of surgery or experience on total energy intake regardless of whether rats were offered only PC or CAF (Fig. 4, Table 3). When we analyzed the first postsurgical day of PC, however, we found that 2NX rats consumed significantly less than SHAM controls when first experiencing PC in the FCM (Fig. 4, Table 4). All groups showed changes in energy intake across days, leading to a main effect of day and an interaction with some other factors but the general lack of differences in body weight and overall energy intake suggests that 2NX rats were healthy and unimpaired, regardless of diet experience. On the surface, the general lack of effect on energy intake may appear to suggest that 2NX does not affect eating, but total intake measures only reveal the outcome of behaviors driven by regulatory processes that ultimately control food intake. In that regard, the specific foods chosen and the manner in which they were consumed must be considered.

**Table 3.** Energy intake statistics

Effects	Energy intake PC	Energy intake CAF
Surgery	*F*_(1,26)_ = 0.956, *p* = 0.337	*F*_(1,26)_ = 1.045, *p* = 0.316
Experience	*F*_(1,26)_ = 0.033, *p* = 0.857	*F*_(1,26)_ = 0.112, *p* = 0.741
Day	***F*_(3,78)_ = 24.246, *p* < 0.001**	***F*_(7,182)_ = 14.46, *p* < 0.001**
S × E	*F*_(1,26)_ = 0.031, *p* = 0.861	*F*_(1,26)_ = 0.033, *p* = 0.858
S × D	***F*_(3,78)_ = 4.188, *p* = 0.008**	***F*_(7,182)_ = 2.609, *p* = 0.014**
D × E	***F*_(3,78)_ = 3.053, *p* = 0.033**	***F*_(7,182)_ = 2.272, *p* = 0.031**
S × D × E	*F*_(3,78)_ = 1.409, *p* = 0.247	*F*_(7,182)_ = 1.409, *p* = 0.204

Results comparing energy intake with 3-way ANOVA during the PC-only period and the CAF phase. PC, powdered chow; CAF, 5-choice custom rodent diet. Effects are defined as follows: S, surgery; E, CAF diet experience; D, day. Statistics with *p* value ≤0.05 are bolded.

**Table 4.** First and last day energy intake statistics

Diet	First day			Last day		
	Surgery	Experience	S × E	Surgery	Experience	S × E
Energy intake-PC	***F*_(1,26)_ = 7.273, *p* = 0.012**	*F*_(1,26)_ = 1.977, *p* = 0.172	*F*_(1,26)_ = 0.012, *p* = 0.915	*F*_(1,26)_ = 0.078, *p* = 0.782	*F*_(1,26)_ = 0.667, *p* = 0.422	*F*_(1,26)_ = 1.528, *p* = 0.227
Energy intake-CAF	*F*_(1,26)_ = 1.435, *p* = 0.242	*F*_(1,26)_ = 1.904, *p* = 0.179	*F*_(1,26)_ = 1.071, *p* = 0.310	*F*_(1,26)_ = 3.536, *p* = 0.071	*F*_(1,26)_ = 0.922, *p* = 0.346	*F*_(1,26)_ = 0.047, *p* = 0.831

Results comparing energy intake with 2-way ANOVA on the first and last day of the PC-only period and of the CAF phase. PC, powdered chow; CAF, 5-choice custom rodent diet. Effects are defined as follows: S, surgery; E, CAF diet experience. Statistics with *p* value ≤0.05 are bolded.

### Lingual gustatory neurotomy increased the proportion of daily energy intake from the HF CAF choices

**Figure 5.**
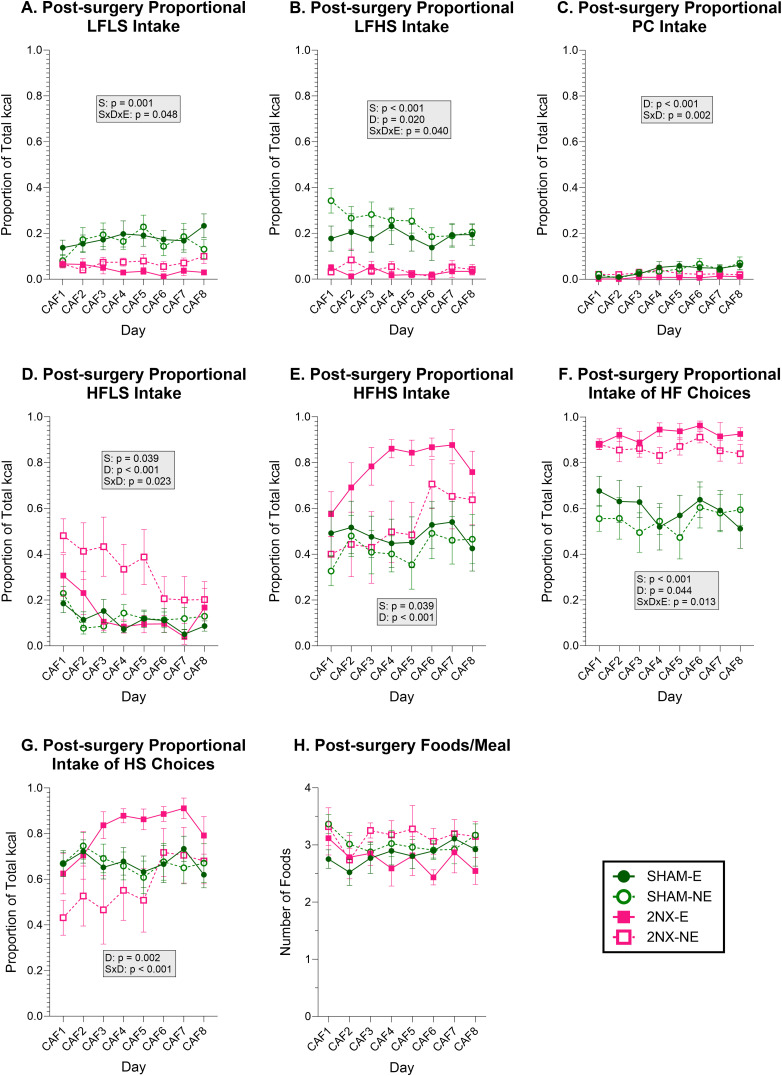
Food choice is altered after 2NX. Data are represented as mean ± SE. Rats that had SHAM surgery are shown as circles and 2NX as squares, and rats with presurgical CAF diet experience (E) are shown with filled symbols and without experience (NE) with open symbols. Significant effects of surgery (S), CAF diet experience (E), and day (D) or interactions between factors are indicated in the shaded boxes for the PC and CAF diet phases. Group sizes: SHAM-E, *n* = 8; 2NX-E, *n* = 6; SHAM-NE, *n* = 8; 2NX-NE, *n* = 8. Statistical details can be found in Tables 5–7. ***A***, Proportion of energy intake from LFLS choice. ***B***, Proportion of energy intake from LFHS choice. ***C***, Proportion of energy intake from PC. ***D***, Proportion of energy intake from HFLS choice. ***E***, Proportion of energy intake from HFHS choice. ***F***, Proportion of energy intake from the HF choices. ***G***, Proportion of energy intake from the HS choices. ***H***, Number of foods chosen per meal.

Although the total calories consumed was comparable between surgical groups, the foods chosen to arrive at these similar energy intakes differed (Fig. 5*A–G* and Table 5). We found main effects of surgery influencing proportional intake of all CAF food choices except PC when rats were offered CAF after surgery. While SHAM rats increased their PC intake over the 8 d testing period, all rats generally ate very little PC while on CAF yielding no main effect of surgery. Rats that had 2NX tended to eat more of the HF food choices and less of the LF food choices compared with SHAM rats (Fig. 5*F*; Tables 6, 7). Intake of each HF food choice by rats that had 2NX was variable, as evidenced by an effect of surgery on HFLS intake on CAF Day 1 but not Day 8 and the opposite pattern for the HFHS choice (Fig. 5*D*,*E*; Table 6). However, the preference for the HF food choices by 2NX rats is clear when the combined proportion of total intake from these two foods is quantified (Fig. 5*F*, Table 7); there was a main effect of surgery on intake of these options on both Day 1 and Day 8 of CAF (Table 6).

**Table 5.** Food intake statistics

Effects	LFLS	LFHS	HFLS	HFHS	PC
Surgery	***F*_(1,26)_ = 13.882, *p* = 0.001**	***F*_(1,26)_ = 22.895, *p* < 0.001**	***F*_(1,26)_ = 4.741, *p* = 0.039**	***F*_(1,26)_ = 4.753, *p* = 0.039**	*F*_(1,26)_ = 3.743, *p* = 0.064
Experience	*F*_(1,26)_ = 0.051, *p* = 0.824	*F*_(1,26)_ = 0.924, *p* = 0.345	*F*_(1,26)_ = 3.716, *p* = 0.065	*F*_(1,26)_ = 2.818, *p* = 0.105	*F*_(1,26)_ = 0.557, *p* = 0.462
Day	*F*_(7,182)_ = 1.754, *p* = 0.099	***F*_(7,182)_ = 2.454, *p* = 0.020**	***F*_(7,182)_ = 7.438, *p* < 0.001**	***F*_(7,182)_ = 5.075, *p* < 0.001**	***F*_(7,182)_ = 4.602, *p* < 0.001**
S × E	*F*_(1,26)_ = 0.537, *p* = 0.470	*F*_(1,26)_ = 0.415, *p* = 0.525	*F*_(1,26)_ = 2.664, *p* = 0.115	*F*_(1,26)_ = 1.039, *p* = 0.317	*F*_(1,26)_ = 0.533, *p* = 0.472
S × D	*F*_(7,182)_ = 1.969, *p* = 0.062	*F*_(7,182)_ = 1.136, *p* = 0.343	***F*_(7,182)_ = 2.392, *p* = 0.023**	*F*_(7,182)_ = 2.366, *p* = 0.025	***F*_(7,182)_ = 3.361, *p* = 0.002**
D × E	*F*_(7,182)_ = 1.018, *p* = 0.420	*F*_(7,182)_ = 0.939, *p* = 0.478	*F*_(7,182)_ = 0.979, *p* = 0.448	*F*_(7,182)_ = 1.239, *p* = 0.284	*F*_(7,182)_ = 0.259, *p* = 0.969
S × D × E	***F*_(7,182)_ = 2.075, *p* = 0.048**	***F*_(7,182)_ = 2.158, *p* = 0.040**	*F*_(7,182)_ = 2.013, *p* = 0.056	*F*_(7,182)_ = 0.794, *p* = 0.593	*F*_(7,182)_ = 0.644, *p* = 0.719

Results comparing intake of each CAF choice with 3-way ANOVA during the CAF phase. CAF choices were high (H) or low (L) in fat (F) and/or sugar (S) and PC, powdered chow. Effects are defined as follows: S, surgery; D, day; E, CAF diet experience. Statistics with *p* value ≤0.05 are bolded.

**Table 6.** Day 1 and Day 8 food intake statistics

Measure	Day 1			Day 8		
	Surgery	Experience	S × E	Surgery	Experience	S × E
LFLS	*F*_(1,26)_ = 2.800, *p* = 0.106	*F*_(1,26)_ = 1.250, *p* = 0.274	*F*_(1,26)_ = 1.220, *p* = 0.280	***F*_(1,26)_ = 8.616, *p* = 0.007**	*F*_(1,26)_ = 0.159, *p* = 0.694	***F*_(1,26)_ = 4.653, *p* = 0.040**
LFHS	***F*_(1,26)_ = 26.374, *p* < 0.001**	*F*_(1,26)_ = 2.963, *p* = 0.097	***F*_(1,26)_ = 4.654, *p* = 0.040**	***F*_(1,26)_ = 23.305, *p* < 0.001**	*F*_(1,26)_ = 0.079, *p* = 0.781	*F*_(1,26)_ = 0.001, *p* = 0.972
PC	*F*_(1,26)_ = 0.164, *p* = 0.689	*F*_(1,26)_ = 3.043, *p* = 0.093	*F*_(1,26)_ = 0.165, *p* = 0.688	***F*_(1,26)_ = 5.941, *p* = 0.022**	*F*_(1,26)_ = 0.156, *p* = 0.696	*F*_(1,26)_ = 0.001, *p* = 0.980
HFLS	***F*_(1,26)_ = 9.673, *p* = 0.005**	*F*_(1,26)_ = 3.295, *p* = 0.081	*F*_(1,26)_ = 1.164, *p* = 0.291	*F*_(1,26)_ = 1.651, *p* = 0.210	*F*_(1,26)_ = 0.417, *p* = 0.524	*F*_(1,26)_ = 0.005, *p* = 0.947
HFHS	*F*_(1,26)_ = 0.863, *p* = 0.361	*F*_(1,26)_ = 4.068, *p* = 0.054	*F*_(1,26)_ = 0.003, *p* = 0.955	***F*_(1,26)_ = 5.806, *p* = 0.023**	*F*_(1,26)_ = 0.147, *p* = 0.704	*F*_(1,26)_ = 0.589, *p* = 0.450
HF CHOICES	***F*_(1,26)_ = 30.340, *p* < 0.001**	*F*_(1,26)_ = 1.610, *p* = 0.216	*F*_(1,26)_ = 1.554, *p* = 0.224	***F*_(1,26)_ = 26.657, *p* < 0.001**	*F*_(1,26)_ = 0.001, *p* = 0.980	*F*_(1,26)_ = 1.754, *p* = 0.197
HS CHOICES	***F*_(1,26)_ = 4.292, *p* = 0.048**	*F*_(1,26)_ = 2.038, *p* = 0.165	*F*_(1,26)_ = 2.034, *p* = 0.166	*F*_(1,26)_ = 1.168, *p* = 0.290	*F*_(1,26)_ = 0.135, *p* = 0.716	*F*_(1,26)_ = 0.948, *p* = 0.339
FOODS/MEAL	*F*_(1,26)_ = 0.426, *p*= 0.520	*F*_(1,26)_ = 2.751, *p* = 0.109	*F*_(1,26)_ = 0.696, *p* = 0.412	*F*_(1,26)_ = 0.618, *p* = 0.439	*F*_(1,26)_ = 2.787, *p* = 0.107	*F*_(1,26)_ = 0.484, *p* = 0.493

Results comparing proportional intake of CAF choices and number of foods per meal with 2-way ANOVA on the first and last day of the CAF phase. CAF choices were high (H) or low (L) in fat (F) and/or sugar (S) and PC, powdered chow. Effects are defined as follows: S, surgery; E, CAF diet experience. Statistics with *p* value ≤0.05 are bolded.

**Table 7.** Statistics for intake of HF or HS foods and foods/meal

Effects	HF combined	HS combined	Foods/meal
Surgery	***F*_(1,26)_ = 28.436, *p* < 0.001**	*F*_(1,26)_ = 0.087, *p* = 0.771	*F*_(1,26)_ = 0.008, *p* = 0.928
Experience	*F*_(1,26)_ = 0.760, *p* = 0.391	*F*_(1,26)_ = 2.785, *p* = 0.107	*F*_(1,26)_ = 2.810, *p* = 0.106
Day	***F*_(7,182)_ = 2.116, *p* = 0.044**	***F*_(7,182)_ = 3.464, *p* = 0.002**	*F*_(7,182)_ = 1.677, *p* = 0.117
S × E	*F*_(1,26)_ = 0.013, *p* = 0.909	*F*_(1,26)_ = 2.768, *p* = 0.108	*F*_(1,26)_ = 0.320, *p* = 0.577
S × D	*F*_(7,182)_ = 1.131, *p* = 0.248	***F*_(7,182)_ = 4.093, *p* < 0.001**	*F*_(7,182)_ = 0.789, *p* = 0.598
D × E	*F*_(7,182)_ = 0.685, *p* = 0.684	*F*_(7,182)_ = 1.212, *p* = 0.298	*F*_(7,182)_ = 0.427, *p* = 0.885
S × D × E	***F*_(7,182)_ = 2.624, *p* = 0.013**	*F*_(7,182)_ = 0.926, *p* = 0.488	*F*_(7,182)_ = 1.492, *p* = 0.173

Results comparing intake of the high-fat (HF) and high sugar (HS) CAF choices and the number of foods per meal with 3-way ANOVA during the CAF phase. Effects are defined as follows: S, surgery; D, day; E, CAF diet experience. Statistics with *p* value ≤0.05 are bolded.

When we make a similar comparison of total intake of the two HS foods (Fig. 5*G*), there is only an effect of surgery on CAF Day 1 (Table 6); if the 8 d period is viewed in total, there is only an effect of day and a surgery × day interaction due to HS intake of SHAM rats remaining relatively consistent while HS intake increased over the CAF phase in 2NX rats (Fig. 5*G*; Tables 6, 7).

Despite differences in the foods chosen among the groups, we found no disparity in the number of foods consumed for each meal (Fig. 5*H*; Tables 6, 7).

### Changes in proportional intake of food choices after GL and CT neurotomy resulted in altered relative macronutrient intake

**Figure 6.**
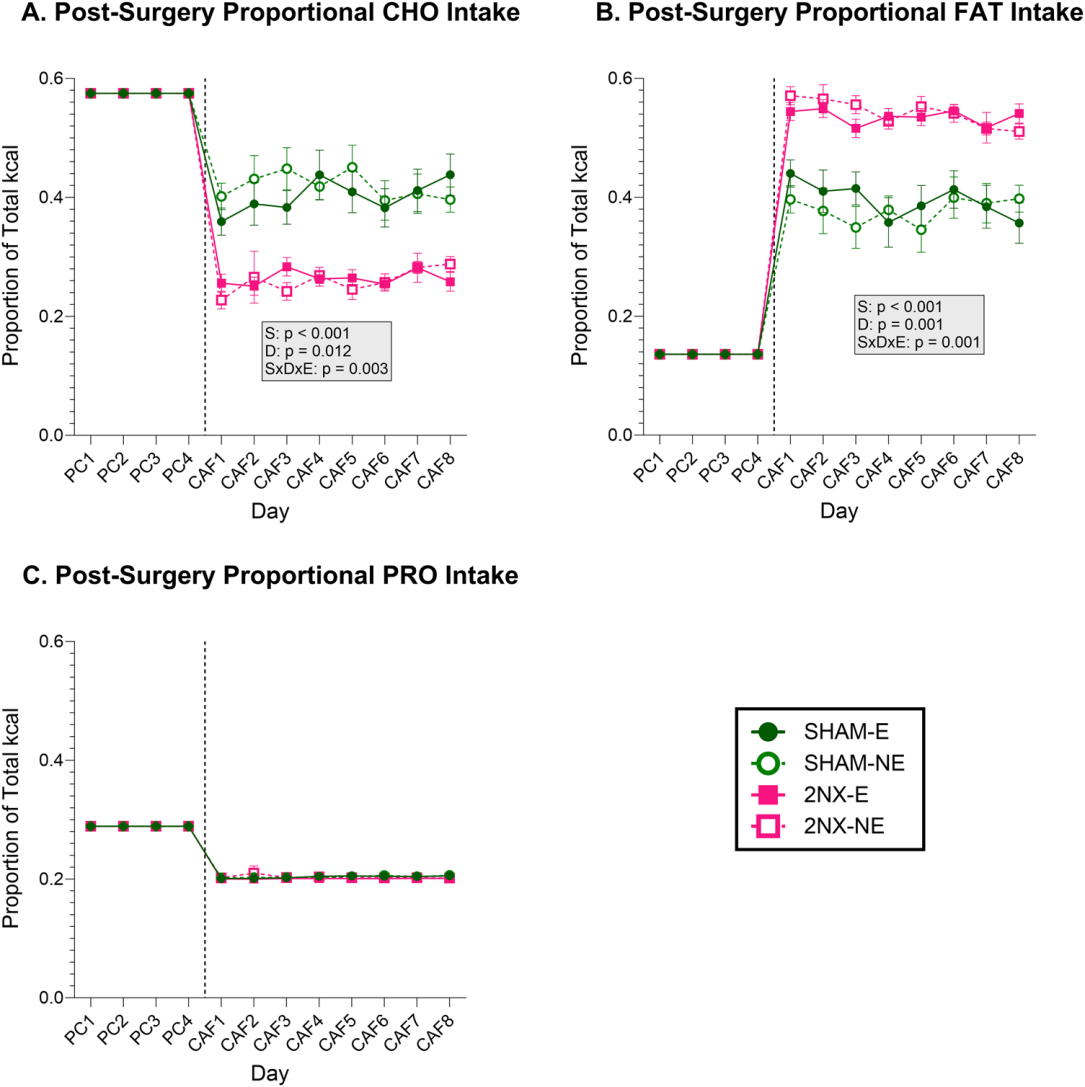
Lingual gustatory nerve transection alters macronutrient intake. Data are represented as mean ± SE. Rats that had SHAM surgery are shown as circles and 2NX as squares, and rats with presurgical CAF diet experience (E) are shown with filled symbols and without experience (NE) with open symbols. The vertical line separates the four PC (powdered chow) days from the eight CAF diet days, which were analyzed separately with three-way ANOVA. Significant effects of surgery (S), CAF diet experience (E), and day (D) or interactions between factors are indicated in the shaded boxes for the PC and CAF diet phases. Group sizes: SHAM-E, *n* = 8; 2NX-E, *n* = 6; SHAM-NE, *n* = 8; 2NX-NE, *n* = 8. Complete statistical details can be found in Tables 8 and 9. ***A***, Proportion of energy intake from all carbohydrate (CHO). ***B***, Proportion of energy intake from FAT. ***C***, Proportion of energy intake from protein (PRO).

Based on earlier work (Treesukosol et al., 2010), we expected 2NX rats to reduce fat intake. However, because 2NX rats consumed more of the combined HF choices and less of the combined LF choices, the fat intake of 2NX rats increased while carbohydrate intake decreased compared with SHAM rats (Fig. 6 and Table 8). Intakes on CAF Day 1 and Day 8 suggest that the effects of the neurotomy on relative fat and carbohydrate intake were consistent over time (Table 9).

**Table 8.** Macronutrient intake statistics

Effects	CHO	FAT	PRO	SUG	NON-SUG CHO
Surgery	***F*_(1,26)_ = 39.237, *p* < 0.001**	***F*_(1,26)_ = 42.293, *p* < 0.001**	*F*_(1,26)_ = 1.074, *p* = 0.310	***F*_(1,26)_ = 17.748, *p* < 0.001**	***F*_(1,26)_ = 13.235, *p* = 0.001**
Experience	*F*_(1,26)_ = 0.071, *p* = 0.791	*F*_(1,26)_ = 0.037, *p* = 0.848	*F*_(1,26)_ = 0.804, *p* = 0.378	*F*_(1,26)_ = 0.222, *p* = 0.641	*F*_(1,26)_ = 0.467, *p* = 0.501
Day	***F*_(7,182)_ = 2.661, *p* = 0.012**	***F*_(7,182)_ = 3.754, *p* = 0.001**	*F*_(7,182)_ = 0.472, *p* = 0.854	*F*_(7,182)_ = 0.863, *p* = 0.537	***F*_(7,182)_ = 2.239, *p* = 0.033**
S × E	*F*_(1,26)_ = 0.205, *p* = 0.655	*F*_(1,26)_ = 0.242, *p* = 0.627	*F*_(1,26)_ = 0.519, *p* = 0.478	*F*_(1,26)_ = 3.933, *p* = 0.058	*F*_(1,26)_ = 1.206, *p* = 0.282
S × D	*F*_(7,182)_ = 0.989, *p* = 0.441	*F*_(7,182)_ = 1.231, *p* = 0.288	*F*_(7,182)_ = 1.163, *p* = 0.326	***F*_(7,182)_ = 3.153, *p* = 0.004**	***F*_(7,182)_ = 3.763, *p* = 0.001**
D × E	*F*_(7,182)_ = 0.548, *p* = 0.797	*F*_(7,182)_ = 0.283, *p* = 0.960	*F*_(7,182)_ = 0.618, *p* = 0.741	*F*_(7,182)_ = 1.136, *p* = 0.342	*F*_(7,182)_ = 0.942, *p* = 0.475
S × D × E	***F*_(7,182)_ = 3.286, *p* = 0.003**	***F*_(7,182)_ = 3.902, *p* = 0.001**	*F*_(7,182)_ = 0.384, *p* = 0.911	*F*_(7,182)_ = 1.839, *p* = 0.082	*F*_(7,182)_ = 1.410, *p* = 0.204

Results comparing the proportion of total energy intake from each macronutrient between groups with 3-way ANOVA. CHO, carbohydrate; SUG, sugar; PRO, protein; S, surgery; E, CAF diet experience; D, day. Statistics with *p* value ≤0.05 are bolded.

**Table 9.** Day 1 and Day 8 macronutrient intake statistics

Measure	DAY 1			DAY 8		
	Surgery	Experience	S × E	Surgery	Experience	S × E
CHO	***F*_(1,26)_ = 48.582, *p* < 0 0.001**	*F*_(1,26)_ = 0.127, *p* = 0.725	*F*_(1,26)_ = 3.147, *p* = 0.088	***F*_(1,26)_ = 36.314, *p* < 0.001**	*F*_(1,26)_ = 0.064, *p* = 0.803	*F*_(1,26)_ = 2.244, *p* = 0.146
FAT	***F*_(1,26)_ = 47.214, *p* < 0.001**	*F*_(1,26)_ = 0.177, *p* = 0.677	*F*_(1,26)_ = 2.986, *p* = 0.096	***F*_(1,26)_ = 38.411, *p* < 0.001**	*F*_(1,26)_ = 0.049, *p* = 0.826	*F*_(1,26)_ = 2.227, *p* = 0.148
PRO	*F*_(1,26)_ = 0.164, *p* = 0.689	*F*_(1,26)_ = 3.043, *p* = 0.093	*F*_(1,26)_ = 0.165, *p* = 0.688	***F*_(1,26)_ = 5.941, *p* = 0.022**	*F*_(1,26)_ = 0.156, *p* = 0.696	*F*_(1,26)_ = 0.001, *p* = 0.980
SUG	***F*_(1,26)_ = 31.766, *p* < 0.001**	*F*_(1,26)_ = 0.279, *p* = 0.602	***F*_(1,26)_ = 7.402, *p* = 0.012**	***F*_(1,26)_ = 10.723, *p* = 0.003**	*F*_(1,26)_ = 0.017, *p* = 0.896	*F*_(1,26)_ = 1.249, *p* = 0.274
NON-SUG CHO	*F*_(1,26)_ = 1.183, *p* = 0.287	*F*_(1,26)_ = 0.065, *p* = 0.802	*F*_(1,26)_ = 1.905, *p* = 0.179	***F*_(1,26)_ = 13.465, *p* = 0.001**	*F*_(1,26)_ = 0.025, *p* = 0.876	***F*_(1,26)_ = 4.446, *p* = 0.045**

Results comparing proportional macronutrient intake with 2-way ANOVA on the first and last day of the CAF phase. CHO, carbohydrate; SUG, sugar; PRO, protein. Effects are defined as follows: S, surgery; E, CAF diet experience. Statistics with *p* value ≤0.05 are bolded.

**Figure 7.**
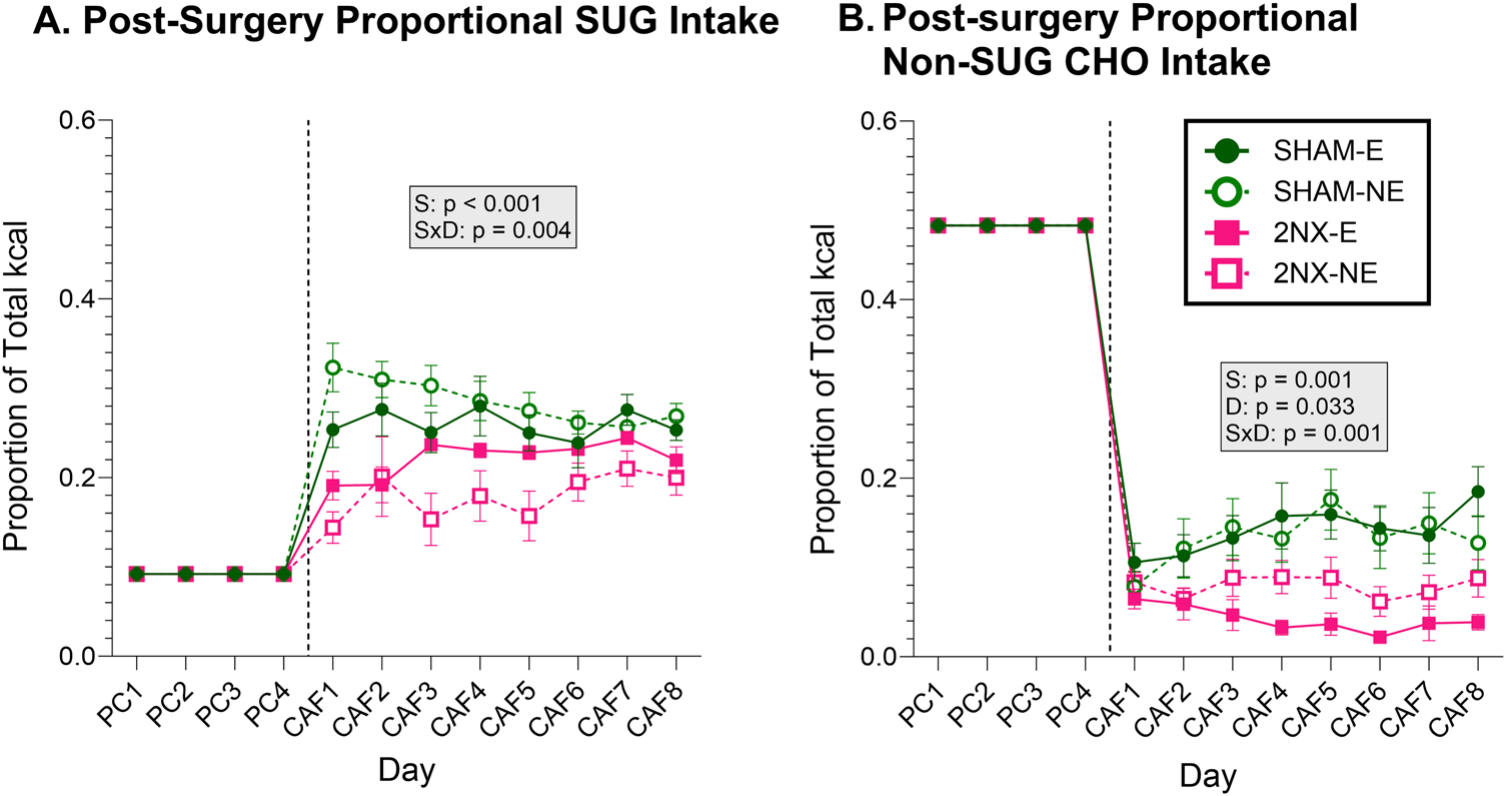
Lingual gustatory nerve transection alters sugar and nonsugar carbohydrate intake. Data are represented as mean ± SE. Rats that had SHAM surgery are shown as circles and 2NX as squares, and rats with presurgical CAF diet experience (E) are shown with filled symbols and without experience (NE) with open symbols. The vertical line separates the four PC (powdered chow) days from the eight CAF diet days, which were analyzed separately with three-way ANOVA. Significant effects of surgery (S), CAF diet experience (E), and day (D) or interactions between factors are indicated in the shaded boxes for the PC and CAF diet phases. Group sizes: SHAM-E, *n* = 8; 2NX-E, *n* = 6; SHAM-NE, *n* = 8; 2NX-NE, *n* = 8. Complete statistical details can be found in Tables 8 and 9. ***A***, Proportion of energy intake from sugar (SUG). ***B***, Proportion of energy intake from nonsugar carbohydrate.

Total carbohydrate intake represents the combined intake of sugar and nonsugar carbohydrate. When analyzing sugar alone, there was a main effect of surgery on intake, with 2NX rats consuming less sugar than rats that had SHAM surgery (Fig. 7*A*, Table 8). The interaction between surgery and day appears to be attributable to 2NX rats tending to increase sugar intake over time while sugar intake of SHAM rats was stable or slightly declining. The effect of surgery on sugar intake was present on CAF Day 1 and Day 8 (Table 9), but there was an additional interaction with experience on Day 1 as there was a greater difference in SUG intake between SHAM and 2NX rats without CAF experience. This finding suggests that the CT and/or GL is needed to drive the initial increases in sugar intake when rats are offered CAF, but over time, it would appear there may be some flavor–nutrient learning occurring with whatever orosensory signals remain. With regard to nonsugar carbohydrate intake, we found main effects of surgery and day and a surgery × day interaction resulting from nonsugar carbohydrate intake of 2NX rats generally remaining stable or decreasing over time while intake of SHAM rats was increasing (Fig. 7*B*, Table 8); a result consistent with our Day 1 and Day 8 analyses (Table 9). Although, as noted above, the effects of 2NX on some food choices changed over days, a comparison of Figures 5 and 7 with Figure 6 shows that regardless of such variability, systematic or otherwise, the neurotomy had a striking effect of shifting the profile of macronutrient intake which remained relatively stable throughout postsurgical CAF testing.

### Lingual gustatory nerve transection changed the way chow and CAF were eaten

**Figure 8.**
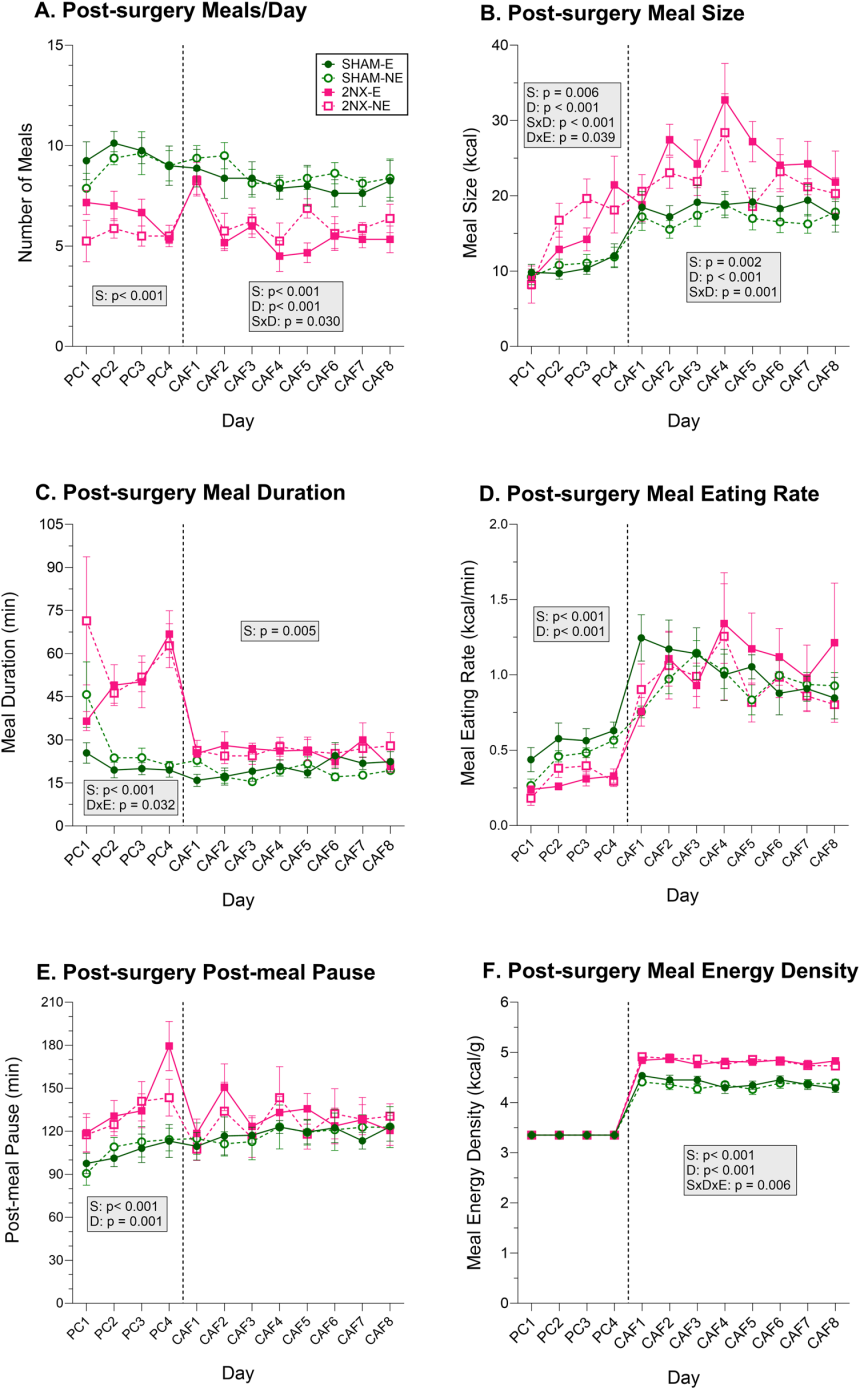
Lingual gustatory nerve transection alters eating patterns. Data are represented as mean ± SE. Rats that had SHAM surgery are shown as circles and 2NX as squares, and rats with presurgical CAF diet experience (E) are shown with filled symbols and without experience (NE) with open symbols. The vertical line separates the four PC (powdered chow) days from the eight CAF diet days, which were analyzed separately with three-way ANOVA. Significant effects of surgery (S), CAF diet experience (E), and day (D) or interactions between factors are indicated in the shaded boxes for the PC and CAF diet phases. Group sizes: SHAM-E, *n* = 8; 2NX-E, *n* = 6; SHAM-NE, *n* = 8; 2NX-NE, *n* = 8. Complete statistical details can be found in Tables 10–13. ***A***, Number of meals per day. ***B***, Meal size as kcal. ***C***, Meal duration (min). ***D***, Meal eating rate (kcal/min). ***E***, Postmeal pause (min). ***F***, Meal energy density (kcal/g).

When rats were placed into the FCM after surgery, effects of the neurotomy on meal patterns were revealed during the initial 4 day PC period (Fig. 8, Tables 10–13). Specifically, compared with SHAM rats, rats that had 2NX consumed fewer, but larger meals at a slower eating rate and spent more time eating with longer pauses between meals. This suggests that while on standard rodent chow, signals from the CT and/or GL are integrated with other signals that contribute to meal termination and motivation to consume PC. That the number of PC meals is reduced and time between meals is increased suggests 2NX rats showed appropriate compensation for the larger meal size via processes that affect meal initiation. As a result, total daily PC intake was relatively unaffected by the transection of the lingual gustatory nerves despite that the way the surgical groups reached that outcome was significantly different.

**Table 10.** Meal number and meal size statistics

Effects	Meals/day-PC	Meals/day-CAF	Meal size (kcal)-PC	Meal size (kcal)-CAF
Surgery	***F*_(1,26)_ = 32.456, *p* < 0.001**	***F*_(1,26)_ = 19.717, *p* < 0.001**	***F*_(1,26)_ = 8.778, *p* = 0.006**	***F*_(1,26)_ = 11.367, *p* = 0.002**
Experience	*F*_(1,26)_ = 1.944, *p* = 0.175	*F*_(1,26)_ = 1.085, *p* = 0.307	*F*_(1,26)_ = 0.284, *p* = 0.599	*F*_(1,26)_ = 1.553, *p* = 0.224
Day	*F*_(3,78)_ = 2.232, *p* = 0.091	***F*_(7,182)_ = 6.912, *p* < 0.001**	***F*_(3,78)_ = 17.338, *p* < 0.001**	***F*_(7,182)_ = 4.546, *p* < 0.001**
S × E	*F*_(1,26)_ = 0.158, *p* = 0.695	*F*_(1,26)_ = 0.043, *p* = 0.838	*F*_(1,26)_ = 0.122, *p* = 0.730	*F*_(1,26)_ = 0.193, *p* = 0.664
S × D	*F*_(3,78)_ = 1.119, *p* = 0.347	***F*_(7,182)_ = 2.279, *p* = 0.030**	***F*_(3,78)_ = 7.699, *p* < 0.001**	***F*_(7,182)_ = 3.550, *p* = 0.001**
D × E	*F*_(3,78)_ = 1.659, *p* = 0.183	*F*_(7,182)_ = 0.569, *p* = 0.781	***F*_(3,78)_ = 2.934, *p* = 0.039**	*F*_(7,182)_ = 1.125, *p* = 0.349
S × D × E	*F*_(3,78)_ = 0.201, *p* = 0.896	*F*_(7,182)_ = 0.761, *p* = 0.621	*F*_(3,78)_ = 1.572, *p* = 0.203	*F*_(7,182)_ = 0.832, *p* = 0.562

Results from 3-way ANOVAs comparing meal parameters while rats had only powdered chow (PC) or CAF. Effects are defined as follows: S, surgery; E, CAF diet experience; D, day. Statistics with *p* value ≤0.05 are bolded.

**Table 11.** Meal duration and meal rate statistics

Effects	Meal duration-PC	Meal duration-CAF	Meal rate (kcal/min)-PC	Meal rate (kcal/min)-CAF
Surgery	***F*_(1,26)_ = 43.510, *p* < 0.001**	***F*_(1,26)_ = 9.391, *p* = 0.005**	***F*_(1,26)_ = 18.387, *p* < 0.001**	*F*_(1,26)_ = 0.054, *p* = 0.819
Experience	*F*_(1,26)_ = 2.773, *p* = 0.108	*F*_(1,26)_ = 0.035, *p* = 0.853	*F*_(1,26)_ = 0.732, *p* = 0.400	*F*_(1,26)_ = 0.650, *p* = 0.428
Day	*F*_(3,78)_ = 1.573, *p* = 0.203	*F*_(7,182)_ = 0.583, *p* = 0.769	***F*_(3,78)_ = 14.979, *p* < 0.001**	*F*_(7,182)_ = 1.798, *p* = 0.090
S × E	*F*_(1,26)_ = 0.000, *p* = 0.997	*F*_(1,26)_ = 0.137, *p* = 0.715	*F*_(1,26)_ = 2.166, *p* = 0.153	*F*_(1,26)_ = 0.022, *p* = 0.884
S ×D	*F*_(3,78)_ = 2.029, *p* = 0.117	*F*_(7,182)_ = 0.972, *p* = 0.453	*F*_(3,78)_ = 2.365, *p* = 0.077	*F*_(7,182)_ = 1.519, *p* = 0.163
D × E	***F*_(3,78)_ = 3.084, *p* = 0.032**	*F*_(7,182)_ = 1.368, *p* = 0.221	*F*_(3,78)_ = 1.833, *p* = 0.148	*F*_(7,182)_ = 0.709, *p* = 0.665
S × D × E	*F*_(3,78)_ = 0.421, *p* = 0.739	*F*_(7,182)_ = 1.736, *p* = 0.103	*F*_(3,78)_ = 1.150, *p* = 0.334	*F*_(7,182)_ = 1.758, *p* = 0.098

Results from 3-way ANOVAs comparing meal parameters while rats had only powdered chow (PC) or CAF. Effects are defined as follows: S, surgery; E, CAF diet experience; D, day. Statistics with *p* value ≤0.05 are bolded.

**Table 12.** Postmeal pause and meal energy density statistics

Effects	Postmeal pause-PC	Postmeal pause-CAF	Meal energy density-CAF
Surgery	***F*_(1,26)_ = 25.750, *p* < 0.001**	*F*_(1,26)_ = 1.196, *p* = 0.284	***F*_(1,26)_ = 46.338, *p* < 0.001**
Experience	*F*_(1,26)_ = 0.392, *p* = 0.537	*F*_(1,26)_ = 0.232, *p* = 0.880	*F*_(1,26)_ = 0.104, *p* = 0.750
Day	***F*_(3,78)_ = 6.802, *p* = 0.001**	*F*_(7,182)_ = 1.459, *p* = 0.185	***F*_(7,182)_ = 4.593, *p* < 0.001**
S × E	*F*_(1,26)_ = 0.807, *p* = 0.377	*F*_(1,26)_ = 0.040, *p* = 0.844	*F*_(1,26)_ = 0.143, *p* = 0.708
S × D	*F*_(3,78)_ = 1.340, *p* = 0.268	*F*_(7,182)_ = 0.879, *p* = 0.525	*F*_(7,182)_ = 1.135, *p* = 0.343
D × E	*F*_(3,78)_ = 0.968, *p* = 0.412	*F*_(7,182)_ = 0.507, *p* = 0.829	*F*_(7,182)_ = 0.209, *p* = 0.983
S × D × E	*F*_(3,78)_ = 0.918, *p* = 0.436	*F*_(7,182)_ = 0.398, *p* = 0.903	***F*_(7,182)_ = 3.662, *p* = 0.001**

Results from 3-way ANOVAs comparing meal parameters while rats had only powdered chow (PC) or CAF. Effects are defined as follows: S, surgery; E, CAF diet experience; D, day. Statistics with *p* value ≤0.05 are bolded.

The overall effects of 2NX on PC meal patterns were not entirely evident on PC Day 1 (Fig. 8, Table 13). On PC Day 1, rats in all groups consumed meals of similar size, meal duration was highly variable, and the eating rate of SHAM rats was somewhat depressed. Potentially, these PC Day 1 effects are at least partly in response to rats being moved into the FCM.

**Figure 9.**
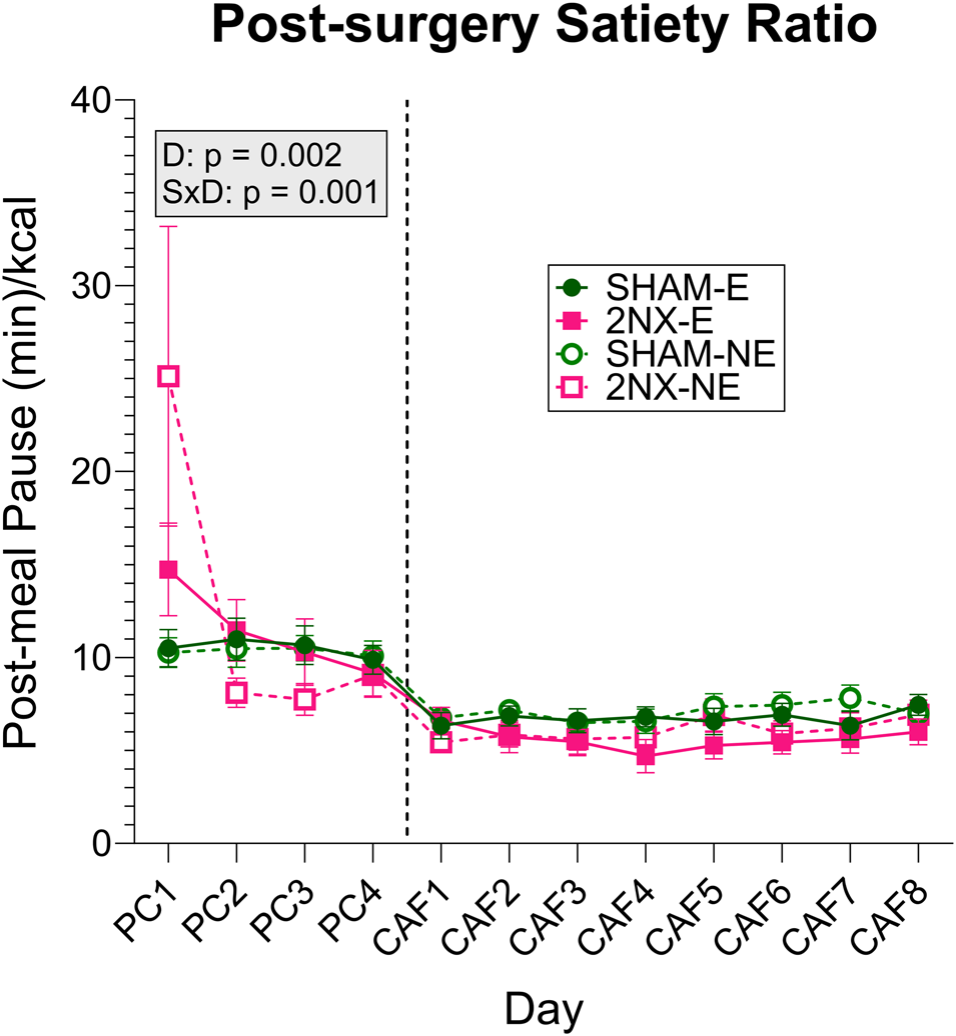
Rats appropriately compensated for their larger meal size after 2NX, as indicated by the post-surgery satiety ratio which was calculated by dividing the post-meal pause (in min) by the kcal consumed during a meal. Data are represented as mean ± SE. Rats that had SHAM surgery are shown as circles and 2NX as squares, and rats with presurgical CAF diet experience (E) are shown with filled symbols and without experience (NE) with open symbols. The vertical line separates the four PC (powdered chow) days from the eight CAF diet days, which were analyzed separately with three-way ANOVA. Significant effects of surgery (S), CAF diet experience (E), and day (D) or interactions between factors are indicated in the shaded boxes for the PC and CAF diet phases. Group sizes: SHAM-E, *n* = 8; 2NX-E, *n* = 6; SHAM-NE, *n* = 8; 2NX-NE, *n* = 8. Complete statistical details can be found in Table 14.

After the 4 d PC period, rats were given the CAF array, and we found changes in meal patterns that for the most part were similar to those found with PC (Fig. 8, Tables 10–13). More precisely, 2NX rats generally continued to consume fewer, larger meals and spend more time eating during meals compared with SHAM rats. This suggests that the processes involved with termination of meals are affected by the transection of the lingual gustatory nerves and that the processes involved with initiation of meals compensate so that energy intake for the day is maintained; this appears to be independent of the solid foods offered because the exact same results occurred when the rats were only offered PC and this assertion is further supported by the lack of effect on satiety ratio during the CAF phase (Fig. 9; Tables 13, 14).

**Table 13.** First and last day meal pattern statistics

Measure	First day			Last day		
	Surgery	Experience	S × E	Surgery	Experience	S × E
Meals/day PC	***F*_(1,26)_ = 7.061, *p* = 0.013**	*F*_(1,26)_ = 3.451, *p* = 0.075	*F*_(1,26)_ = 0.093, *p* = 0.762	***F*_(1,26)_ = 23.858, *p* < 0.001**	*F*_(1,26)_ = 0.013, *p* = 0.910	*F*_(1,26)_ = 0.013, *p* = 0.910
Meals/day CAF	*F*_(1,26)_ = 1.134, *p* = 0.297	*F*_(1,26)_ = 0,0.071 *p* = 0.792	*F*_(1, 26)_ = 0.139, *p* = 0.712	***F*_(1, 26)_ = 7.706, *p* = 0.010**	*F*_(1, 26)_ = 0,0.434 *p* = 0.516	*F*_(1,26)_ = 0.268, *p* = 0.609
Meal size PC	*F*_(1,26)_ = 0.333, *p* = 0.569	*F*_(1,26)_ = 0.158, *p* = 0.694	*F*_(1,26)_ = 0.002, *p* = 0.969	***F*_(1,26)_ = 9.836, *p* = 0.004**	*F*_(1,26)_ = 0.488, *p* = 0.491	*F*_(1,26)_ = 0.408, *p* = 0.529
Meal size CAF	*F*_(1,26)_ = 0.776, *p* = 0.387	*F*_(1,26)_ = 0.015, *p* = 0.902	*F*_(1,26)_ = 0.529, *p* = 0.474	*F*_(1,26)_ = 2.079, *p* = 0.161	*F*_(1,26)_ = 0.033, *p* = 0.858	*F*_(1,26)_ = 0.191, *p* = 0.666
Meal dur PC	*F*_(1,26)_ = 1.791, *p* = 0.192	*F*_(1,26)_ = 4.033, *p* = 0.055	*F*_(1,26)_ = 0.284, *p* = 0.599	***F*_(1,26)_ = 65.815, *p* < 0.001**	*F*_(1,26)_ = 0.053, *p* = 0.820	*F*_(1,26)_ = 0.258, *p* = 0.616
Meal dur CAF	***F*_(1,26)_ = 6.209, *p* = 0.019**	*F*_(1,26)_ = 2.405, *p* = 0.133	*F*_(1,26)_ = 1.351, *p* = 0.256	*F*_(1,26)_ = 1.113, *p* = 0.301	*F*_(1,26)_ = 0.404, *p* = 0.531	*F*_(1,26)_ = 2.456, *p* = 0.129
Meal rate PC	***F*_(1,26)_ = 6.315, *p* = 0.019**	***F*_(1,26)_ = 4.156, *p* = 0.052**	*F*_(1,26)_ = 1.014, *p* = 0.323	***F*_(1,26)_ = 30.715, *p* < 0.001**	*F*_(1,26)_ = 1.018, *p* = 0.322	*F*_(1,26)_ = 0.088, *p* = 0.769
Meal rate CAF	*F*_(1,26)_ = 1.706, *p* = 0.206	*F*_(1,26)_ = 1.726, *p* = 0.00	***F*_(1,26)_ = 5.930, *p* = 0.022**	*F*_(1,26)_ = 0.408, *p* = 0.529	*F*_(1,26)_ = 0.754, *p* = 0.393	*F*_(1,26)_ = 1.678, *p* = 0.207
Postmeal pause PC	***F*_(1,26)_ = 5.551, *p* = 0.026**	*F*_(1,26)_ = 0.164, *p* = 0.689	*F*_(1,26)_ = 0.077, *p* = 0.784	***F*_(1,26)_ = 15.202, *p* = 0.001**	*F*_(1,26)_ = 2.036, *p* = 0.166	*F*_(1,26)_ = 2.286, *p* = 0.143
Postmeal pause CAF	*F*_(1,26)_ = 0.007, *p* = 0.933	*F*_(1,26)_ = 0.079, *p* = 0.781	*F*_(1,26)_ = 0.698, *p* = 0.411	*F*_(1,26)_ = 0.045, *p* = 0.834	*F*_(1,26)_ = 0.181, *p* = 0.674	*F*_(1,26)_ = 0.203, *p* = 0.656
Meal enrg density	***F*_(1,26)_ = 40.517, *p* < 0.001**	*F*_(1,26)_ = 0.179, *p* = 0.676	*F*_(1,26)_ = 2.446, *p* = 0.130	***F*_(1,26)_ = 43.116, *p* < 0.001**	*F*_(1,26)_ = 0.003, *p* = 0.954	*F*_(1,26)_ = 2.306, *p* = 0.141
Satiety ratio-PC	***F*_(1,26)_ = 4.446, *p* = 0.045**	*F*_(1,26)_ = 1.257, *p* = 0.272	*F*_(1,26)_ = 1.378, *p* = 0.251	*F*_(1,26)_ = 0.740, *p* = 0.397	*F*_(1,26)_ = 0.003, *p* = 0.958	*F*_(1,26)_ = 0.021, *p* = 0.886
Satiety ratio-CAF	*F*_(1,26)_ = 0.914, *p* = 0.348	*F*_(1,26)_ = 0.461, *p* = 0.503	*F*_(1,26)_ = 2.100, *p* = 0.159	*F*_(1,26)_ = 1.371, *p* = 0.252	*F*_(1,26)_ = 0.111, *p* = 0.741	*F*_(1,26)_ = 1.138, *p* = 0.296

Results comparing meal parameters with 2-way ANOVA on the first and last day of the PC and CAF phases. Effects are defined as follows: S, surgery; E, CAF diet experience. Statistics with *p* value ≤0.05 are bolded.

**Table 14.** Satiety ratio statistics

Effects	Satiety ratio-PC	Satiety ratio-CAF
Surgery	*F*_(1,26)_ = 1.064, *p* = 0.312	*F*_(1,26)_ = 3.820, *p* = 0.062
Experience	*F*_(1,26)_ = 0.095, *p* = 0.760	*F*_(1,26)_ = 0.538, *p* = 0.470
Day	***F*_(3,78)_ = 5.632, *p* = 0.002**	*F*_(7,182)_ = 1.605, *p* = 0.136
S × E	*F*_(1,26)_ = 0.182, *p* = 0.673	*F*_(1,26)_ = 0.012, *p* = 0.915
S × D	***F*_(3,78)_ = 5.688, *p* = 0.001**	*F*_(7,182)_ = 0.611, *p* = 0.746
D × E	*F*_(3,78)_ = 2.023, *p* = 0.118	*F*_(7,182)_ = 1.376, *p* = 0.218
S × D × E	*F*_(3,78)_ = 1.996, *p* = 0.121	*F*_(7,182)_ = 1.377, *p* = 0.218

Results from 3-way ANOVAs comparing satiety ratio while rats had only powdered chow (PC) or CAF. Effects are defined as follows: S, surgery; E, CAF diet experience; D, day. Statistics with *p* value ≤0.05 are bolded.

Unlike PC, however, for which eating rates differed between surgical groups, we did not find any effects on meal eating rate during the 8 d CAF phase (Fig. 8*D*, Table 11). Although the comparison of within-meal eating rates calculated from multifood meals should be done with caution, at the very least, our findings suggest that 2NX and SHAM rats were similarly motivated to consume CAF.

Because the 2NX rats were consuming more of the most energy dense choices from the CAF array compared with SHAM rats, their meal energy density was elevated, and except for minor differences (as indicated by the significant surgery × day × experience interaction), this was largely independent of experience (Fig. 8*F*; Tables 12, 13) and persisted across the CAF phase (Table 13).

## Discussion

### Rats that had lingual gustatory nerve transection did not show signs of impaired health or ability to consume food

Transection of the lingual gustatory nerves caused only a short-lived decrease in body weight and hardly affected energy intake in rats, no matter the available diet, even though 2NX denervates ∼70% of taste buds. The similarities in weight trajectories and daily energy intake of our surgical groups suggest eating was not impaired by 2NX; this is further bolstered by prior work indicating that 2NX does not alter licking (St. John et al., 1994; Spector et al., 1996). It is additionally notable that Aslani et al. (2015) found that rats maintained on a high-fat diet reduced their intake during a chronic mild stress protocol. Although those rats did not have choices and we did not take any direct measurements of stress, the increased fat intake and lack of an effect on daily energy intake after the neurotomy suggests that our rats were not experiencing chronic mild stress after the nerve transection surgeries. Altogether our assertion that the ability to ingest and the health of 2NX rats was not unlike SHAM rats is well supported. Importantly, the similarities in food intake and body weight gain trajectories between 2NX and SHAM rats lead us to conclude that the differences between groups were not merely a mass action type of effect causing general impairments in eating and likely reflect other more specific factors at play as discussed below.

### The CT and/or GL is important for food selection and regulation of macronutrient intake

Although total energy intake was unaffected, compared with SHAM controls, the 2NX rats chose foods in different proportions leading to a dramatically different macronutrient intake profile during the postsurgical CAF period. Specifically, 2NX rats consumed more of the high-fat foods and less of the low-fat choices, leading to their much higher relative fat intake over the test period. In one earlier study, rats that had CTX + GLX + GSPX ultimately did not differ from SHAM rats in response to a high-fat solid food (Dotson et al., 2012). However, the triple-transected rats in that study persistently ate less and weighed less than controls while our groups were similar in intake and body weight so drawing comparisons with the current study is equivocal at best. Other earlier studies transected only the CT or GL and found that both single neurotomies blunted intake and behavioral responsiveness to fat emulsions (Stratford et al., 2006; Gaillard et al., 2008; Pittman, 2010; Treesukosol et al., 2010), making our findings surprising. There are at least two possibilities accounting for the disparity between our results and those from prior studies that cut only the CT or GL.

First, some property of the fats offered could be critical. The prior single nerve cut studies presented relatively pure fats in liquid form while we offered fats as components of nutritionally complex solid foods. In particular, food sources offered here were 6–42% fat by weight with energy densities that ranged between 3.35 and 5.41 kcal/g and the nonfat mass consisting of carbohydrate or protein. In contrast, the prior single nerve transection studies presented stimuli ranging from almost no fat to 16% fat with estimated energy densities <1.5 kcal/g and the nonfat mass being mostly water. Thus, the physical state of the stimuli (liquid vs solid) as well as its energy density and composition can all be critical factors in determining how the loss of the lingual gustatory nerves will affect ingestive behavior.

Second, the prior studies of the effect of lingual gustatory neurotomy on behavioral responsiveness to fat emulsions only focused on a single nerve, either the CT or the GL. In our study, both nerves were transected in combination. We, therefore, cannot rule out that the greater gustatory deafferentation in our work contributed to the apparent interpretive disparity with the outcomes in studies transecting only the CT or GL.

Schreiber et al. (2020) transected both the CT and GL and, similar to the single nerve cut studies, found that intake of a high-fat diet was reduced in obesity-resistant but not obesity-prone rats. Although there were a variety of methodological differences between the two studies, one critical distinction is that the rats in the Schreiber et al. (2020) study did not have food choices, and we have recently published data showing that available choices affect the response to cafeteria diet options (Cawthon and Spector, 2023).

It is interesting to note that fat detection thresholds, to our knowledge, have not been measured in rats after lingual gustatory nerve transection. It is, therefore, possible that a change in fat taste sensitivity could play a role in our findings. This possibility should be tested using rigorous psychophysical methods in a future experiment.

### Maintenance of eating patterns requires input from the CT and/or glossopharyngeal nerve

Meal pattern analysis revealed that after 2NX, rats consumed more energy at each meal but ate fewer meals each day compared with their SHAM counterparts, regardless of whether only chow or multiple palatable food choices were available. That these changes occurred irrespective of the available diet implies that information delivered by the CT and/or GL is directly integrated with other signals (e.g., from vagal afferents) that ultimately lead to meal termination, which was delayed when the input from the lingual gustatory nerves was absent. There is evidence in humans that oral contact time contributes to satiation (de Graaf and Kok, 2010), and this information could be provided by the CT and/or GL. Perhaps these signals play a role in a central metering of caloric content before the food is swallowed; such metering based on oral sensory input has been proposed in the literature (Mook, 1963; Mook et al., 1983). Smith et al. (1988) found that while CTX alone increased PC meal duration and reduced PC eating rate, it did not change the number of meals or amount consumed per day. Notwithstanding minor experimental differences between that study and the work conducted here, the findings from Smith et al. (1988) suggest that the increase in meal size we observed required transection of the GL, but it remains to be tested whether GLX alone or transection of both lingual gustatory nerves is necessary to recapitulate the effects on meal size we observed.

### Presurgical diet experience had minimal effects on postsurgical CAF eating behavior

In research investigating the effects of gustatory nerve transection on basic taste function, there is evidence that experience with the stimuli prior to surgery influences postsurgical outcomes. For example, Markison et al. (1999) showed that rats without pre-GLX quinine experience had blunted licking avoidance in response to quinine during postsurgical testing, in contrast to the more minor effect of GLX reported in rodents with presurgical quinine exposure (St. John et al., 1994). Another study found that the GSP was sufficient for rats to relearn a salt discrimination task they learned prior to 2NX (Blonde et al., 2010). Findings such as these led us to expect that, potentially, presurgical CAF experience might lessen the effects of gustatory neurotomy on food selection and ingestive behavior when tested after surgery. To our surprise, the effect of presurgical diet exposure was minimal on measures we analyzed, and inspection of Tables 3–14 reveals that the main effects of, or interactions with, CAF experience were few. Of the behavioral outcomes we examined, sugar intake is perhaps the clearest example where surgical groups diverged based on presurgical cafeteria diet exposure. Studies of basic taste-guided behavior after gustatory nerve transection have demonstrated only mildly impaired affective responses to sucrose after 2NX in rats that have had presurgical testing (Spector et al., 1996). In the present experiment, sugar intake of 2NX rats that had presurgical CAF experience, compared with 2NX rats that did not, matched SHAM levels sooner. In rats with presurgical CAF experience, perhaps the signals transmitted via the GSP, which in rats is exceptionally responsive to sugar stimuli (Nejad, 1986; Travers and Norgren, 1991), are sufficient to drive intake of the HS choices. In contrast, in rats without presurgical CAF exposure, the lack of a full complement of taste receptors apparently has a greater impact on HS choices until sufficient experience increases preference perhaps through flavor–nutrient learning mediated by the remaining sensory signals; a progressive increase was also observed across postsurgical test days in 2NX rats that had presurgical CAF exposure.

### Limitations

The current study includes limitations. First, the subjects were male so we do not know if similar effects would be observed in females. Second, because both the CT and GL were transected, we cannot determine the relative contribution that the loss of each nerve had to the effects described here. Third, although we would note that *ad libitum* food access better emulates human eating conditions, we are unable to report whether there is any physiological state dependency on the response of rats to a cafeteria diet after lingual gustatory nerve transection because we only tested rats under *ad libitum* feeding conditions. Fourth, although we consider the loss of lingual taste signals as the most parsimonious explanation for the behavioral consequences of 2NX, these nerves also contribute to other functions. The CT has afferents that respond to temperature (Ogawa et al., 1968; Breza et al., 2006) and mechanical stimulation (Matsuo et al., 1995; Yokota and Bradley, 2017) and the GL provides the somatosensory innervation for the posterior tongue (Bradley et al., 1997). These nerves also supply some autonomic innervation to some salivary glands (Hellekant and Hagstrom, 1974; Gurkan and Bradley, 1987) and lingual vasculature (Hellekant, 1977). That said, the lingual branch of the trigeminal nerve, which is the primary conduit of somatosensory input from the anterior tongue to the brain, was left intact. This nerve branch also provides partial parasympathetic innervation of the submandibular gland in rats (Hellekant and Kasahara, 1973; Young and Van Lennep, 1978). The reduced PC eating rate displayed by 2NX rats could possibly result from even mildly compromised oral secretions since PC is very dry. Regardless of whether these changes are fully the result of lost taste input, the function of the lingual gustatory nerves in nutrition has been largely overlooked in the literature and our robust results testify to their critical role in the control of food selection, relative macronutrient intake, and meal-taking behavior.

### Conclusion

To our knowledge, we are the first to conduct detailed eating analysis after histologically verified lingual gustatory nerve transection in rats with more than two food choices. By employing our state-of-the-art FCM with five food choices, we revealed that 2NX shifts food preference toward choices higher in fat, altering macronutrient intake, and were accompanied by meal pattern changes, all without affecting total energy consumption. These findings show that under conditions in which rats have choices between multiple complex nutrient sources, signals from the CT and/or GL significantly contribute to processes that control food selection, regulation of relative macronutrient intake, and meal termination but are not necessary for the maintenance of long-term energy balance. Additional research is needed to determine the role of the individual nerves in our results and investigate what influence, if any, is provided by the GSP, which innervates taste buds of the palate. Future research should also aim to identify the downstream brain regions and properties of this signal integration. Our findings clearly highlight the importance of analyzing the fine details of behavior (i.e., meal patterns) and not just its outcome (i.e., total intake).

## Synthesis

Reviewing Editor: Masha Prager-Khoutorsky, McGill University Decisions are customarily a result of the Reviewing Editor and the peer reviewers coming together and discussing their recommendations until a consensus is reached. When revisions are invited, a fact-based synthesis statement explaining their decision and outlining what is needed to prepare a revision will be listed below. The following reviewer(s) agreed to reveal their identity: NONE. Note: If this manuscript was transferred from JNeurosci and a decision was made to accept the manuscript without peer review, a brief statement to this effect will instead be what is listed below.

Synthesis of Reviews:

While reviewers agreed that the manuscript has the potential to advance the field, they indicated that there are some critical points that have to be fully addressed.

The most significant issues are:

1. The authors should provide convincing verification and quantifications of nerve transections, by presenting appropriate histological analysis

2. The authors should adequately address the contradictions between their findings and previous studies, explaining discrepancies with previous studies

3 The authors should address potential influence of stress, by adding some additional discussion on how they considered stress did or did not impact their findings.

---------------

Reviewer#1:

The current manuscript by Cawthon et al. aims to investigate how dual chorda tympani (CT ) and glossopharyngeal (GL) nerves transections (2NX) affect behavioral responses to chow and a 5-choice cafeteria diet (CAF) in male rats. For this, they had four experimental groups: 1. rats with dual CT and GL nerve transections -2NX; 2.SHAM controls; 3. Pre-exposed to CAF 2NX; 4. Pre-exposed to CAF SHAM.

The major findings of the current study are:

1. 2NX and Pre-exposed to CAF 2NX rats increased their intake of high-fat chow, as compared to SHAM and Pre-exposed to CAF SHAM controls.

2. 2NX and Pre-exposed to CAF 2NX rats decreased their carbohydrate intake, as compared to SHAM and Pre-exposed to CAF SHAM controls.

3. There were no major effects on body weight or caloric energy consumption.

4. 2NX Pre-exposed to CAF 2NX rats ate fewer and larger meals.

Comments

1. Although the authors report to have verified and quantified nerve transections - histology (e.g., images of fungiform papillae following 0.5% methylene blue staining), and quantification should be provided for all groups for rigor and reproducibility.

2. Do the dual CT and GL nerve transections affect fat or sugar detection? If so, how? If not, how then do the authors explain the changes in food selection they observed in the 2NX rats?

3. Are the effects observed in the dual CT and GL nerve transections (i.e., increases in high-fat chow intake and reduced carbohydrate intake) concentration or state-dependent?

4. Also, how do the authors explain the discrepancies between their findings showing that the 2NX rats increase fat intake and the findings from other studies, such as Schreiber et al., 2020 - showing that CT and GL nerve transections result in decreased high-fat food intake in obesity-prone rats, and has no effect on high-fat food intake in obesity-resistant rats?

5. How do the authors rule out the effects of stress after 2NX as a potential explanation for their findings? Indeed, previous studies have shown that rats exposed to mild chronic stress increase their high-fat consumption (Aslani et al., 2015). As such, the increased high-fat consumption in the 2NX groups could be due to the mild chronic stress of undergoing CT and GL nerve transections.

6. In lines 36-37, the authors write, " Eating rates implied that, compared to SHAM, 2NX rats were equally motivated to consume CAF, but less motivated to eat chow." Based on their findings and experimental design, the authors cannot conclude this as they are not testing for "motivation" per se. Hunger and reward can influence motivation, but eating and food intake can also be dissociated from motivation (e.g., Randall et al., 2012).

------

Reviewer#2:

I believe this manuscript does provide advances in the field. The gustatory system plays a crucial role in guiding ingestive behavior, and much work has been dedicated to understanding this role in basic taste functions. However, there is a lack of work that investigates how information from taste receptors and gustatory afferents influences ingestive behavior, particularly regarding daily macronutrient consumption and food selection and intake patterns. This manuscript is an original research article that uses original data to address a novel hypothesis to provide insight on this understudied aspect of gustatory signaling. The authors hypothesize that transection of the chorda tympani and glossopharyngeal nerves, that provide a large portion of afferent information from taste receptors to the brain, will alter food choices and intake patterns. Gustatory neurotomy has been shown to modulate basic taste functions, in addition to unclear effects on food selection, intake patterns, and nutrient selection. Here, the authors performed gustatory neurotomy that was histologically verified to confirm nerve transection and paired this surgical approach with the author's custom 5-item Food Choice Monitor to determine the effect of removing information from these nerves on food intake and nutrient selection. In addition, they considered experience of the diet prior to gustatory neurotomy on these effects. This approach to investigate the hypothesis is novel by employing the histological verification of the transection of gustatory nerves with detailed eating analysis using the author's Food Choice Monitor. This work highlights the importance of looking at other details of ingestive behavior, such as meal patterns, to understand the role of information from gustatory afferents play in guiding such behavior. Further, the results contribute to the knowledge that information from the gustatory system plays a critical role in regulating ingestive behavior and provide novel evidence that expands our understanding on how this occurs through food choice and intake patterns, providing the foundation for future work to discern the role of the individual gustatory nerves and neural pathways involved.

Comments:

1. Consider providing reference images for 2NX histological verification reflecting 2NX vs SHAM rats

2. Statistical tables referenced in some figure legends (e.g. Figure 7) do not appear to correspond to that figure or match what is noted in the results section for corresponding figures and statistical tables

3. Line 587: typo in Table 1 legend - (F) and).

4. Lines 676-677: Figure 4 legend mentions vertical line separating PC and CAF however no such line appears in the figure
